# Anticancer potential of phytochemicals derived from mangrove plants: Comprehensive mechanistic insights

**DOI:** 10.1002/fsn3.4318

**Published:** 2024-07-10

**Authors:** Raihan Chowdhury, Md. Shimul Bhuia, Md. Sakib Al Hasan, Shadid Hossain Snigdha, Sadia Afrin, Dietrich Büsselberg, Solomon Habtemariam, Eda Sönmez Gürer, Javad Sharifi‐Rad, Afaf Ahmed Aldahish, Nursulu Аkhtayeva, Muhammad Torequl Islam

**Affiliations:** ^1^ Department of Pharmacy Bangabandhu Sheikh Mujibur Rahman Science and Technology University Gopalganj Bangladesh; ^2^ Phytochemistry and Biodiversity Research Laboratory BioLuster Research Center Gopalganj Bangladesh; ^3^ Pharmacy Discipline Khulna University Khulna Bangladesh; ^4^ Weill Cornell Medicine‐Qatar, Education City Qatar Foundation Doha Qatar; ^5^ Pharmacognosy Research & Herbal Analysis Services UK Kent UK; ^6^ Faculty of Pharmacy, Department of Pharmacognosy Sivas Cumhuriyet University Sivas Turkey; ^7^ Department of Biomedical Sciences College of Medicine, Korea University Seoul Republic of Korea; ^8^ Department of Pharmacology, College of Pharmacy King Khalid University Abha Saudi Arabia; ^9^ Department of Biodiversity and Bioresources of Al‐Farabi Kazakh National University Almaty Kazakhstan

**Keywords:** anticancer phytochemicals, cancer, mangrove plants, molecular mechanisms

## Abstract

Cancer is a collection of illnesses characterized by aberrant cellular proliferation that can infiltrate or metastasize to distant anatomical sites, posing a notable threat to human well‐being due to its substantial morbidity and death rates worldwide. The potential of plant‐derived natural compounds as anticancer medicines has been assessed owing to their favorable attributes of few side effects and significant antitumor activity. Mangrove plants and their derived compounds have been scientifically shown to exhibit many significant beneficial biological activities, such as anti‐inflammatory, immunomodulatory, antioxidant, neuroprotective, cardioprotective, and hepatoprotective properties. This study summarized mangrove plants and their derived compounds as potential anticancer agents, with an emphasis on the underlying molecular mechanisms. To explore this, we gathered data on the preclinical (in vivo and in vitro) anticancer effects of mangrove plants and their derived compounds from reputable literature spanning 2000 to 2023. We conducted thorough searches in various academic databases, including PubMed, ScienceDirect, Wiley Online, SpringerLink, Google Scholar, Scopus, and the Web of Science. The results demonstrated that mangrove plants and their derived compounds have promising anticancer properties in preclinical pharmacological test systems through various molecular mechanisms, including induction of oxidative stress and mitochondrial dysfunction, cytotoxicity, genotoxicity, cell cycle arrest, apoptosis, autophagy, antiproliferative, antimetastatic, and other miscellaneous actions. Upon thorough observation of the pertinent information, it is suggested that mangrove plants and their derived chemicals may serve as a potential lead in the development of novel drugs for cancer therapy.

## Graphical abstract

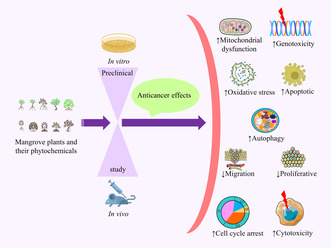

## 1 INTRODUCTION

Cancer is a pathological state characterized by the aberrant and unregulated proliferation of cells inside the human body. The mass of tissue formed due to this unregulated proliferation is commonly referred to as a malignant tumor. Tumor cells have a destructive behavior toward healthy tissues and vital organs (Bhat et al., 2023; Bhuia, Chowdhury, et al., 2023a). Likewise, cancer may originate in a specific anatomical region and then metastasize to distant sites throughout the body through a process called metastasis (Peart, 2017). Multiple variables, including physical, chemical, environmental, metabolic, and genetic factors, contribute to the initiation and progression of various forms of cancer (Kumari et al., 2022). According to research published by the World Health Organization (WHO), the global mortality rate of cancer in 2020 amounted to approximately 10 million deaths, or nearly one‐sixth of the total deaths globally (https://www.who.int/news‐room/fact‐sheets/detail/cancer).

In South‐East Asia alone, the WHO recorded in 2020 2,252,981 newly reported cases and 1,444,528 deaths attributed to cancer. The most commonly reported types of cancer among new cases were breast, cervical, and lung cancers (The Lancet Regional Health‐Southeast Asia, 2023). The United States was expected to have 1,958,310 new cancer cases and 609,820 cancer‐related deaths in 2023. The incidence of prostate cancer had a yearly rise of 3% from 2014 to 2019, after a reduction in the last two decades. This upward trend resulted in an extra 99,000 new cases.

Conversely, the incidence trends for other types of cancer were comparatively more favorable for men than women (Siegel et al., 2023). The increased susceptibility of males to many forms of cancer is often attributed to their heightened exposure to environmental and behavioral variables known to be carcinogenic, such as smoking. However, recent research has shown that other disparities contribute significantly to this phenomenon (Jackson et al., 2022; Rawla, 2019). Potential factors might include height, exposure to endogenous hormones, and immunological function and reaction (Choi et al., 2019; Klein & Flanagan, 2016). Cancer now ranks among the primary contributors to morbidity and mortality on a global scale (Lindert et al., 2021).

Treatment options used in cancer include surgical intervention, radiation, chemotherapy, and immunotherapy, with chemotherapeutics being a prominent modality within this spectrum. However, in practical application, treatments are often seen as lacking effectiveness, despite demonstrating advancements in therapeutic outcomes (Bhuia et al., 2023b; Debela et al., 2021). Chemotherapy medications are considered relatively nonselective agents due to their ability to have harmful effects on normal cells alongside their intended anticancer action (Senapati et al., 2018). Patients may have shown persistent infertility, alopecia, oral ulcerations, cardiac anomalies, hematopoietic impairment, and cardiovascular irregularities. Bone marrow toxicities lead to anemia and a reduced ability to effectively battle pathogenic infections (Nurgali et al., 2018; Wang & Tepper, 2021). Nausea and vomiting are among the most feared adverse effects experienced by cancer patients under chemotherapy treatment (Mustian et al., 2008). In order to mitigate these adverse effects, conventional botanical remedies or novel anticancer drugs derived from medicinal plants might be considered (Desai et al., 2008; Greenwell & Rahman, 2015).

Traditional medicines, such as Chinese traditional medicine, Ayurveda, Korean traditional medicine, and Unani, have been widely used since ancient times. The pharmaceutical formulations in these preparations are derived from natural sources and are used to treat several human maladies (Fabricant & Farnsworth, 2001; Yuan et al., 2016). Mangrove plants have significant potential as sources of medicine for those living in native areas and adjacent settlements (Dey et al., 2021). Mangrove plants are found worldwide in 123 tropical and subtropical nations, with 84 distinct kinds of total mangrove species (Cerri et al., 2022; Nebula et al., 2013). Mangrove plants possess diverse bioactive compounds found among different plant components, including leaves, stems, bark, fruit, and roots (Patra & Thatoi, 2011). The mangrove plants have several bioactivities, such as antioxidant, anti‐inflammatory, antidiabetic, and anti‐obesity properties (Qaed et al., 2023).

Additionally, mangrove plants and their derived phytochemicals suppress cancer cell growth via a variety of mechanisms, including apoptosis (Chaudhry et al., 2021), inhibiting proliferation effect (Parthiban et al., 2023), inducing cytotoxicity (Μatsumoto et al., 2020), cell cycle arrest (Sahai et al., 2020), inducing oxidative stress (Neumann et al., 2015), inducing autophagy (Majumder et al., 2020), inhibiting DNA repair (Samarakoon et al., 2017), and metastatic effect (Huang et al., 2016). The primary goal of the present study was to evaluate the anticancer abilities of mangrove plants and their compounds in great detail. In addition, we seek to investigate the molecular processes that give rise to mangrove plants and their phytochemical anticancer properties, focusing on their possible application in cancer treatment, which could provide valuable insights for future investigations and the development of novel therapeutic approaches.

## 2 METHODOLOGY

### 2.1 Search strategy

We included data from 2000 to December, 2023 in this study, and the literature was chosen by searching electronic databases, such as PubMed, ScienceDirect, SpringerLink, Wiley Online, Web of Science, and Scopus with the terms “Mangrove plants,” then paired with “Cancer,” “Tumor,” “Pathophysiology of cancer,” “Anticancer activity,” “Anti‐proliferation activity,” “Apoptotic effect,” “Oxidative stress,” “Protective effect,” “Cytotoxic activity,” “Genotoxic activity,” “Carcinogenesis,” “Anti‐angiogenic effect,” “Antitumor activity,” “Human cancer,” “Biological activities,” “Biological evaluation,” “Medicinal use,” “Pharmacology,” “Pharmacological effects,” “Pharmacological activities,” “Toxicity,” “*In vivo* studies,” or “*In vitro* studies.” There were no language restrictions. The papers were thoroughly reviewed, with information on the botanical names, extracts or compounds, dose, concentration, test system, hypothesized anticancer effect mechanism, and overall conclusion provided. The following are the inclusion and exclusion criteria.

### 2.2 Inclusion and exclusion criteria

Inclusion: (a) Anticancer activity of true mangroves and mangrove‐associated mangroves in tropical and subtropical areas worldwide. (b) In vitro, ex vivo, and in vivo studies carried out with or without using various experimental animals and their derived tissues or cells. (c) Mangrove plant crude extracts or fractions or isolated compounds from the plants or their laboratories as semisynthetic, synthetic preparations, or derivatives. (d) Studies with preliminary or advanced phytoconstituents and/or pharmacological investigations. (e) Studies of mangrove plant‐isolated compounds' anticancer activities and chemical features. (f) Studies with or without hypothesizing bimolecular mechanisms. Exclusion: (i) Mangrove‐associated fungi and their derived compounds. (ii) Studies exhibited duplicate data and/or titles and abstracts that did not meet the inclusion criteria. (iii) Other studies on mangrove plants uncover the current topic. (iv) Papers written in languages other than English. (v) Studies without full text available. (vi) Case reports, letters, editorials, and commentaries.

## 3 FINDINGS AND DISCUSSION

### 3.1 Mangrove and mangrove plants

Mangroves are forest ecosystems that exhibit salt tolerance and are mostly distributed in the intertidal portions of tropical and subtropical areas worldwide. Their distribution is predominantly limited to the geographic belt between 30° N and 30° S or 25° N and 30° S of the equator (Lontsi et al., 2023; MacNae, 1969; Valiela et al., 2001). Mangroves, which are found in 112 nations and territories, cover about 25% of the global shoreline, spanning a total area of 181,000 square kilometers (Spalding et al., 1997; Sridhar, 2009). Mangroves are highly productive ecosystems that thrive abundantly in coastal locations, river estuaries, and backwater regions. Their development is contingent upon the presence of a muddy substrate with varied depths and consistencies. They inhabit low‐lying regions that experience frequent tidal flooding (Patra & Thatoi, 2011).

The mangrove habitat is characterized by its dynamic and harsh nature, and as a result, mangrove species have developed diverse adaptations to effectively withstand and thrive in these challenging environmental circumstances (Naskar & Palit, 2015). The subterranean tissues of all plants need oxygen for the process of respiration. However, in the specific ecological setting of mangroves, the availability of oxygen in the soil is severely restricted or absent altogether. Consequently, the uptake of oxygen from the atmosphere is required by the mangrove root system. Mangrove species possess unique above‐ground roots known as breathing roots, or pneumatophores, which have a specific function in this context. Certain species have roots that resemble slender pencils or pegs, while others possess roots that bear a resemblance to a knee‐like structure. The roots possess a multitude of pores that facilitate the ingress of oxygen into the subterranean tissues. Buttress roots in some plant species have the dual purpose of facilitating gas exchange and providing structural reinforcement to the tree (Das et al., 2014; Srikanth et al., 2016). Certain species of mangroves have a characteristic root structure whereby the roots deviate from the main stem and branches, extending into the soil at a considerable distance from the central axis, similar to the growth pattern seen in banyan trees. The term “stilt roots” is attributed to their physical characteristics and their primary function of providing structural support. Additionally, these roots possess many pores that facilitate the ingress of ambient oxygen into the root system (Ohira et al., 2013; Scholander et al., 1955). The presence of salty water and unconsolidated saline soil with limited or absent oxygen creates an unfavorable setting for seed germination and establishment. In order to surmount this challenge, mangrove species use a unique method of reproduction, often referred to as vivipary. This reproductive strategy involves the germination and subsequent development of seeds into seedlings while they remain connected to the parent tree. The term often used to refer to these seedlings is “propagules,” which engage in photosynthesis while remaining connected to the parent tree. The parental tree provides water and essential nutrients. The objects in question exhibit buoyancy, allowing them to remain afloat in water for a certain duration before establishing anchorage in appropriate soil (Selvam, 2007; Shi et al., 2005).

**FIGURE 1:**
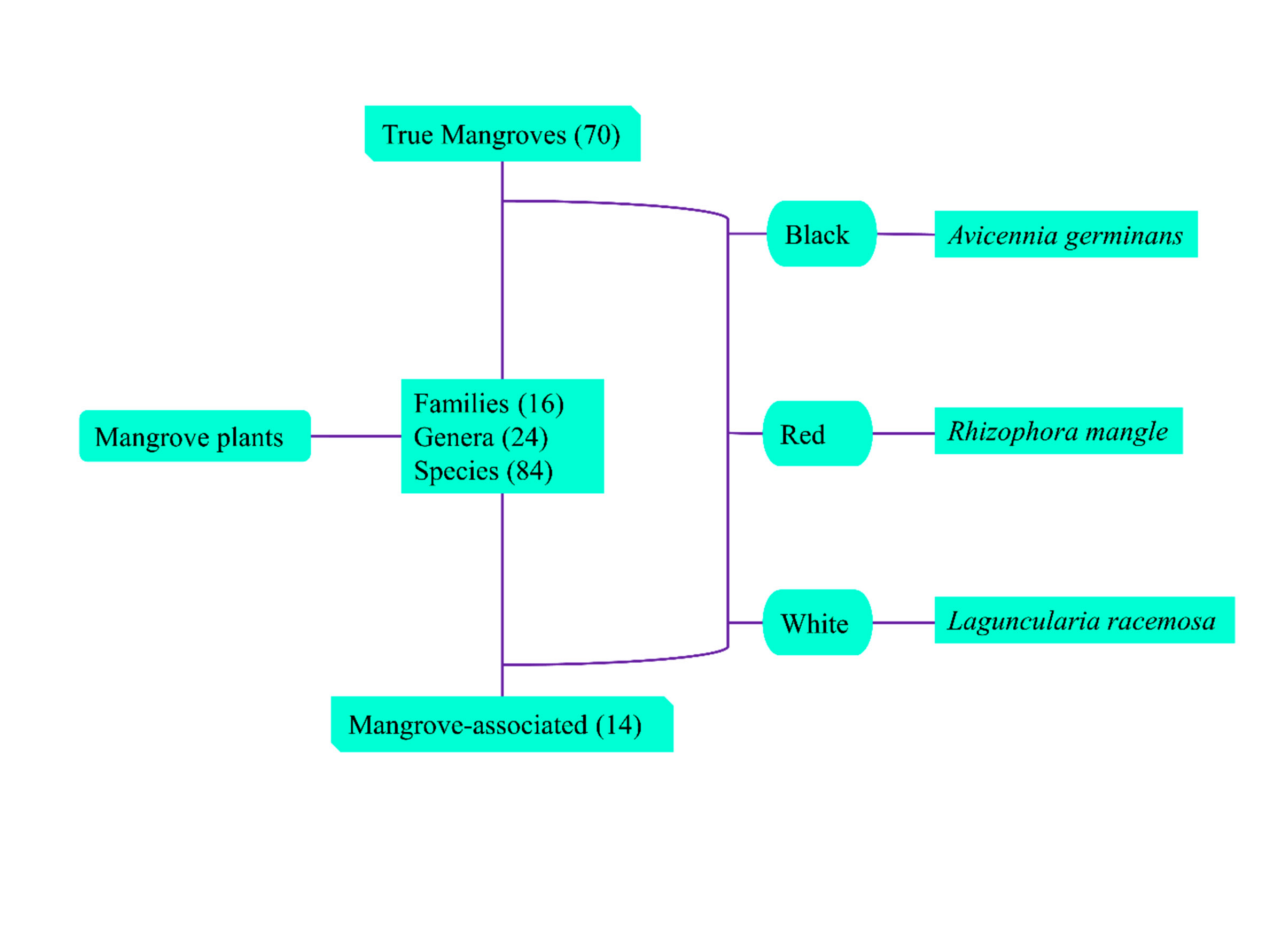
Different classes of mangrove plants.

The classification of mangroves includes three distinct kinds of tropical wetland trees that thrive in the coastal regions of central and southern Florida, where they are often found along estuaries' shorelines. The three aforementioned species, namely black mangrove (*Avicennia germinans*), red mangrove (*Rhizophora mangle*), and white mangrove (*Laguncularia racemosa*), are indigenous to the state of Florida (Spier et al., 2016). Table 1 presents the distinguishing characteristics of several components of mangrove plants, including fruits, leaves, and roots of three prevalent species of mangroves (Nabeelah Bibi et al., 2019). Additionally, mangrove plants are generally classified into two distinct categories, namely true mangroves and mangrove‐associated mangroves (Wang et al., 2018). True mangrove species only thrive inside the mangrove ecosystem and do not expand their presence into terrestrial plant communities. These species possess morphological, physiological, and reproductive adaptations that enable them to thrive in salty, waterlogged, and anaerobic conditions (Alappatt, 2008). The distribution of true mangrove plants is mostly limited to intertidal mangrove habitats, whereas mangrove‐associated plants are found at the landward edge of mangrove ecosystems or in terrestrial marginal zones that experience irregular high tides (Mitra et al., 2021; Sur et al., 2016). Research data showed that the global mangrove plant population consists of a total of 84 species, distributed among 24 genera and 16 families. Among these, 70 species are classified as true mangroves, while the other 14 species are categorized as mangrove‐associated (Nebula et al., 2013; Wu et al., 2008). Different classes of mangrove plants are represented in Figure 1.

**TABLE 1:** Distinguishing characteristics of several components of mangrove plants, including fruits, leaves, and roots of three prevalent species of mangroves.

Plant parts	Black mangrove	Red mangrove	White mangrove	Reference
Fruits	Teardrop‐shaped	Cigar‐shaped	Smallest in size	Nabeelah Bibi et al. (2019)
Leaves	Gray in color in bottom surface less shiny, pointy	Very pointy green on both sides, very shiny	Shiny on both sides, round	
Roots	Roots grow against gravity from the soil surface, pneumatophores or pencil‐like roots	Rhizophores or arc‐shaped prop roots, roots come out of the stem and grow downward to end in the soil	–	

### 3.2 Active phytochemicals of mangrove plants

Phytochemicals include a diverse group of naturally occurring chemical compounds found in plants. Phytochemicals may be categorized into primary and secondary metabolites depending on their role in plant metabolism (Rabizadeh et al., 2022). Primary metabolites are essential components for sustaining plant life. These include amino acids, proteins, carbohydrates, lipids, and nucleic acids. In contrast, secondary metabolites refer to the residual plant compounds synthesized by cells through metabolic processes distinct from the core metabolic pathways (Hu et al., 2020; Hussain et al., 2012). These chemical constituents often possess antiviral, antifungal, and antibiotic properties, conferring plant defense against pathogenic organisms. The utilization of plant secondary metabolites in traditional medicine has persisted throughout the history of mankind owing to their notable biological efficacy. Plant chemicals are credited with medicinal capabilities (Hussein & El‐Anssary, 2019; Jadimurthy et al., 2023; Riaz et al., 2023). Mangrove plants possess a diverse array of secondary compounds, such as saponins, alkaloids, glycosides, flavonoids, terpenes, and polyphenols (Dahibhate et al., 2019; Lakshmanan et al., 2013; Nurdiani et al., 2012). These compounds exhibit various beneficial effects, including anti‐inflammatory (Van Thanh et al., 2019), immunomodulatory (Das et al., 2022), antioxidant (Dahibhate et al., 2020), neuroprotective (Zhou et al., 2016), cardioprotective (Vinoth et al., 2019), hepatoprotective (Gnanadesigan et al., 2017), and anticancer activities (Sahai et al., 2020).

**FIGURE 2:**
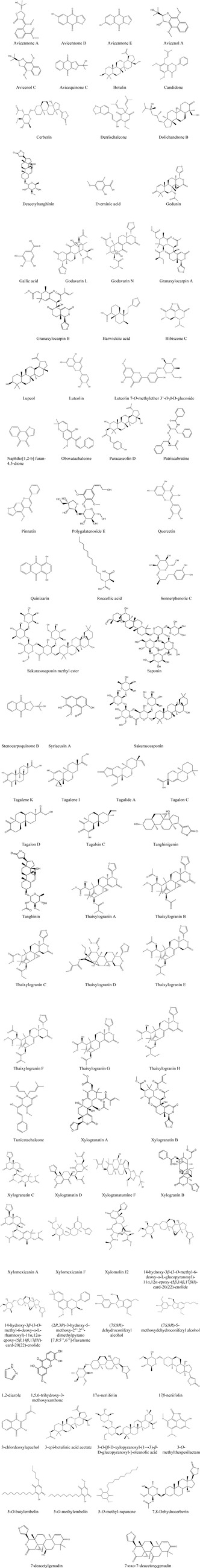
Anticancer phytochemicals isolated from the mangrove medicinal plants.

The mangrove phytochemicals, including 1,2‐diazole (Vinod Prabhu et al., 2018), Gedunin (Sahai et al., 2020), Naphtho[1,2‐b] furan‐4,5‐dione (Chien et al., 2019), Tagalide A (Zhang, Yang, Liu, et al., 2018c), and Tagalsin C (Neumann et al., 2015), were isolated from different plants. All these compounds mediate oxidative stress and mitochondrial dysfunction in cancer cells. Additionally, the compounds (2*R*,3*R*)‐3‐hydroxy‐5‐methoxy‐2″,2″‐dimethylpyrano [7,8:5″,6″]‐flavanone (Decharchoochart et al., 2014), (7*S*,8*R*)‐5‐methoxydehydroconiferyl alcohol, (7*S*,8*R*)‐dehydroconiferyl alcohol (Nguyen, Pham, et al., 2015b), 1,5,6‐trihydroxy‐3‐methoxyxanthone (Nguyen, Bui, et al., 2015a), 17*α*‐neriifolin, 17*β*‐neriifolin (Laphookhieo et al., 2004; Syarifah et al., 2011), 3‐chlorodeoxylapachol (Jones et al., 2005), 3‐*O*‐[*β*‐D‐xylopyranosyl‐(1 → 3)‐*β*‐D‐glucopyranosyl‐]‐oleanolic acid (Tran et al., 2022), 5‐*O*‐butylembelin, 5‐*O*‐methylembelin, 5‐*O*‐methyl‐rapanone (Li et al., 2020), 7,8‐dehydrocerberin (Cheenpracha et al., 2004), 7‐deacetylgenudin, 7‐oxo‐7‐deacetoxygenudin (Pudhom et al., 2009), Avicennone A, Avicennone D, Avicennone E, Avicenol A, Avicenol C, Avicequinone C (Han et al., 2007), Candidone (Decharchoochart et al., 2014), Cerberin (Laphookhieo et al., 2004), Deacetyltanghinin (Cheenpracha et al., 2004), Derrischalcone (Decharchoochart et al., 2014), Dolichandrone B (Nguyen et al., 2018), Tagalene K (Ni et al., 2018), Everninic acid (Mishra et al., 2017), Gedunin (Uddin et al., 2007), Godavarin L, Godavarin N (Zhang, Shi, Liu, et al., 2018b), Granaxylocarpin A, Granaxylocarpin B (Yin et al., 2007), Harwickiic acid (Ba Vinh et al., 2018), Hibiscone C (Μatsumoto et al., 2020), Luteolin 7‐*O*‐methylether 3′‐*O*‐*β*‐D‐glucoside (Sharaf et al., 2000), Obovatachalcone (Decharchoochart et al., 2014), Paracaseolin D (Gong et al., 2017), Patriscabratine (Uddin et al., 2012), Pinnatin (Saraphon et al., 2017), Polygalatenoside E (Nguyen, Bui, et al., 2015a), Polyisoprenoid (Sari et al., 2018), Quinizarin (Sachithanandam et al., 2022a), Roccellic acid (Mishra et al., 2017), Sakurasosaponin, Sakurasosaponin methyl ester (Vinh et al., 2019), Sonnerphenolic C (Nguyen, Pham, et al., 2015b), Stenocarpoquinone B (Han et al., 2007), Syriacusin A (Μatsumoto et al., 2020), Tagalene I, Tagalons C and D (Zhang, Li, Shen, & Wu, 2018d), Tagalsin C, Tanghinin (Cheenpracha et al., 2004), Tetracosane (Uddin et al., 2012), Thaixylogranins A–H (Liao et al., 2017), Tunicatachalcone (Decharchoochart et al., 2014), Xylogranatin A, Xylogranatin B, Xylogranatin C, Xylogranatin D (Pudhom et al., 2009; Yin et al., 2006), Xylogranatumine F (Zhou et al., 2014), Xylogranin B (Toume et al., 2013), and Xylomolin J2 (Zhang et al., 2018d) were shown to be cytotoxic to cancer cells. Furthermore, phytochemicals, such as Gedunin (Sahai et al., 2020), Luteolin (Momtazi‐Borojeni et al., 2013), Naphtho[1,2‐b] furan‐4,5‐dione (Chien et al., 2019), and Tagalsin C (Neumann et al., 2015), induce DNA damage, resulting in genotoxic effects in different types of cancer cells. Additionally, compounds like 1,2‐diazole (Vinod Prabhu et al., 2018), 3‐*O*‐methylthespesilactam (Li et al., 2013), Gedunin (Sahai et al., 2020), Neriifolin (Zhao et al., 2011), Polyisoprenoid (Illian et al., 2019; Sari et al., 2018), Tagalide A (Zhang, Yang, Liu, et al., 2018c), and Tagalsin C (Neumann et al., 2015) induce cancer cell cycle arrest. The studies demonstrated that the compounds, including 1,2‐diazole (Vinod Prabhu et al., 2018), 3‐*O*‐methylthespesilactam (Li et al., 2013), Gedunin (Sahai et al., 2020), Hibisceusone A, Hibisceusone B, Hibisceusone C (Chen et al., 2022), Lupeol (Eldohaji et al., 2021), Luteolin (Momtazi‐Borojeni et al., 2013), Naphtho[1,2‐b] furan‐4,5‐dione (Chien et al., 2019), Neriifolin (Zhao et al., 2011), Patriscabratine (Uddin et al., 2012), Polyisoprenoid (Illian et al., 2019; Sari et al., 2018), Tagalide A (Zhang et al., 2018c), Tagalsin C (Neumann et al., 2015), Tanghinigenin (Wang et al., 2010), and Tetracosane (Uddin et al., 2012), showed apoptotic effects on cancer cells. On the other hand, the chemicals including 14‐hydroxy‐3*β*‐(3‐*O*‐methyl‐6‐deoxy‐*α*‐L‐glucopyranosyl)‐11*α*,12*α*‐epoxy‐(5*β*,14*β*,17*βH*)‐card‐20(22)‐enolide, 14‐hydroxy‐3*β*‐(3‐*O*‐methyl‐6‐deoxy‐*α*‐L‐rhamnosyl)‐11*α*,12*α*‐epoxy‐(5*β*,14*β*,17*βH*)‐card‐20(22)‐enolide, 17*β*‐neriifolin (Chang et al., 2000), 3‐epi‐betulinic acid acetate (He et al., 2007), 3‐*O*‐methylthespesilactam (Li et al., 2013), Avicennone A, Avicennone D, Avicennone E, Avicenol A, Avicenol C, Avicequinone C (Han et al., 2007), Botulin (He et al., 2007), Everninic acid (Mishra et al., 2017), Gallic acid (GA) (Sachithanandam, Parthiban, et al., 2022b), Gedunin (Sahai et al., 2020), Hibisceusone A, Hibisceusone B, Hibisceusone C (Chen et al., 2022), Luteolin (Momtazi‐Borojeni et al., 2013), Naphtho[1,2‐b] furan‐4,5‐dione (Chien et al., 2019), Neriifolin (Zhao et al., 2011), Polyisoprenoid (Sari et al., 2018), Quercetin (Sachithanandam et al., 2022b), Roccellic acid (Mishra et al., 2017), Saponin (Yang et al., 2018), Stenocarpoquinone B (Han et al., 2007), Tanghinigenin (Wang et al., 2010), Xylomexicanin A (Shen et al., 2009), and Xylomexicanin F (Wu, Qing, et al., 2014b) showed antiproliferative effect on cancer cells. Finally, the compound Gedunin showed antimetastatic activity in cancer cells (Sahai et al., 2020). All phytochemicals from mangrove plants are presented in Figure 2 based on their anticancer properties.

### 3.3 Anticancer effect: Underlying molecular mechanisms

#### 3.3.1 Induction of oxidative stress and mitochondrial dysfunction

Oxidative stress is caused by an excess of reactive oxygen species (ROS) and the dysfunction of protective antioxidant mechanisms (Li et al., 2021; Montezano & Touyz, 2012). This imbalance can be triggered by the deregulation of mitochondrial activity, giving rise to an excessive generation of ROS (Ježek et al., 2018). As ROS accumulates beyond tolerable levels, it becomes a primary driver of cell apoptosis, cellular senescence, heightened autophagy, and disruptions in mitochondrial function (Nieh et al., 2022). Oxidative stress frequently manifests when cells undergo apoptosis following exposure to anticancer drug treatments (Murray & Mirzayans, 2020; Rezatabar et al., 2019).

Studies revealed that mangrove plants and their active phytochemicals induce oxidative stress and mitochondrial dysfunction in cancer treatment (preclinical study). Silver nanoparticles (AgNPs) synthesized from Avicennia marina also exhibit amplified anticancer activity against A549 lung cancer cells by elevating ROS levels and subsequently leading to a decrease in mitochondrial membrane potential (Tian et al., 2020; Varunkumar et al., 2020). The studies further demonstrated that Avicennia marina extract and its isolated compound, Naphtho[1,2‐b] furan‐4,5‐dione could increase ROS and decrease mitochondrial membrane potential in cancer cell lines, ultimately resulting in autophagy and cancer cell death via apoptosis (Chien et al., 2019; Esau et al., 2015). The compound Gedunin, isolated from *Xylocarpus granatum*, triggers dose‐dependent ROS generation, damaging DNA and causing programmed cell death in ovarian cancer (Sahai et al., 2020). Furthermore, compounds Tagalsin C and Tagalide A obtained from the *Ceriops tagal* have increased ROS generation in different cancer cells (Neumann et al., 2015; Zhang, Yang, Liu, et al., 2018c). Additionally, 1,2‐diazole decreased mitochondrial membrane potential in A549 lung cancer cells obtained from *Rhizophora apiculata* (Vinod Prabhu et al., 2018). According to Majumder et al. (2020), the chloroform fraction of *Chaetomorpha brachygona* induces increased autophagy by raising ROS levels and decreasing mitochondrial membrane potential in cervical cancer cell lines (Majumder et al., 2020).

#### 3.3.2 Cytotoxicity

Cytotoxicity screening is an experimental technique for assessing a chemical's impact and toxicity on a standard cell line. The proposed or identified lead chemical compound should be experimentally assessed on different human cell lines to confirm its anticancer property while showing no adverse effects on normal cells (Brunner et al., 2006; Rai & Lall, 2021). New anticancer drug development requires extensive cytotoxicity testing (Setiawati et al., 2022). It is essential to measure cytotoxic effects that are dependent on time and concentration (Docea et al., 2016). The mechanisms of cytotoxic drugs in cancer cells include inhibition of cell division and DNA destruction (Saha, 2022). The wingless‐related integration site (Wnt)/β‐catenin pathway regulates cellular proliferation and differentiation (Morris et al., 2022). However, the abnormal activation of the Wnt/β‐catenin signaling pathway results in the formation of tumors (Aoki et al., 2022; Khalaf et al., 2018). Cytotoxic drugs inhibit the excessive activation of these pathways (Doo et al., 2020).

**FIGURE 3:**
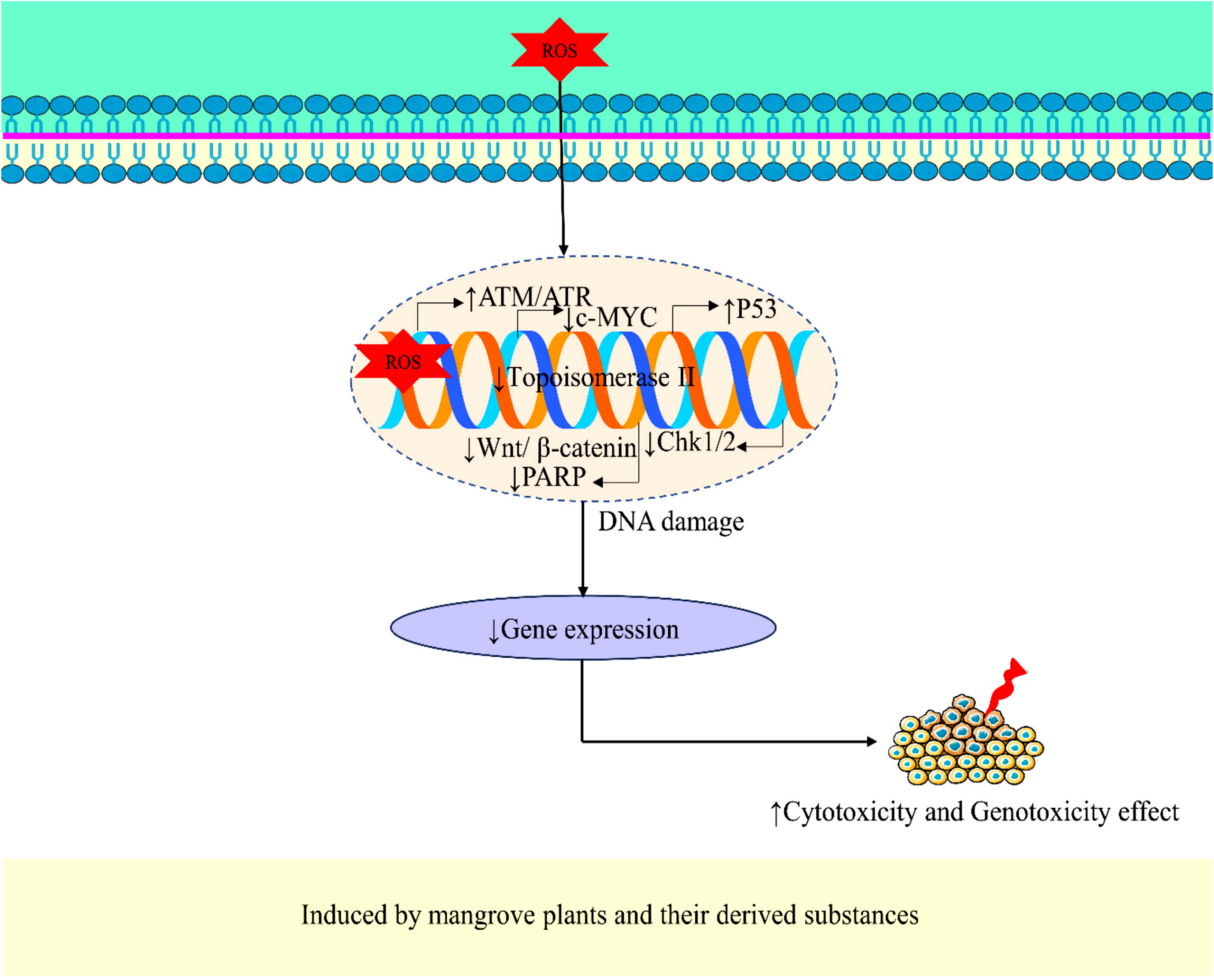
Possible cytotoxic and genotoxic effects of mangrove plants and their derived compounds (↑, increase; ↓, decrease; ATM, Ataxia telangiectasia *mutated*; ATR, Acidosis tubulare renale; Chk1/2, checkpoint kinases Chk1 and Chk2; p53, Tumor protein p53; PARP, Poly adenosine diphosphate‐ribose polymerase; ROS, Reactive oxygen species).

Mangrove plants and their derived phytochemicals may make excellent candidates for research into possible anticancer effects because of their cytotoxic capabilities. The phytochemical Paracaseolin D was extracted from *Sonneratia paracaseolaris* and exhibited cytotoxicity on the A549 cancer cell line with an IC_50_ (half‐maximal inhibitory concentration) value of 1.89 μM. Additionally, Polyisoprenoids from *Nypa fruticans* showed revealed cytotoxicity on colon cancer cells (WiDr and 3T3) through the downregulation of Cyclin D1 and cell cycle (at G0–G1 phase) as well as B‐cell lymphoma 2 (Bcl‐2). The IC_50_ values were 180.2 and 397.7 μg/mL for two cancer cells (Gong et al., 2017; Sari et al., 2018). Several studies revealed that the extract and isolated compounds, such as Avicennone A, Stenocarpoquinone B, Avicequinone C, Avicennone D, Avicennone E, Avicenol A/C, and Luteolin 7‐*O*‐methylether 3′‐*O*‐*β*‐D‐glucoside from *Avicennia marina*, exhibited cytotoxicity in different types (L‐929, K562, HeLa, BT‐20, HL‐60, and HepG2) of cancer cells, leading to the inhibition of cancer proliferation (Han et al., 2007; Karami et al., 2012; Sharaf et al., 2000; Sohaib et al., 2022). The cytotoxic effects of the mangrove plant *Xylocarpus granatum* have also been seen using its extract and purified compounds, namely Granaxylocarpin A, Granaxylocarpin B, Xylogranatin A, Xylogranatin B, Xylogranatin C, Xylogranatumine F, Xylogranatin D, Gedunin, 7‐oxo‐7‐deacetoxygenudin, 7‐deacetylgenudin, and Xylogranin B. These compounds have demonstrated the ability to hinder the growth of cancer cells with IC_50_ values of 6.3–18.83 μM. The study confirmed that Xylogranin B demonstrated cytotoxic effects in SW480 and HCT116 cancer cells by suppressing the activity of Wnt/β‐catenin and T‐cell factor (TCF)/β‐catenin signaling pathways, as well as downregulating the expression of c‐myc and peroxisome proliferator‐activated receptor delta (PPARδ) with an IC_50_ of 48.9 nM (Pudhom et al., 2009; Toume et al., 2013; Uddin et al., 2007; Yin et al., 2006, 2007; Zhou et al., 2014). Furthermore, several studies revealed that many compounds extracted from *Ceriops tagal*, including Tagalene I, Tagalon C, and Tagalon D, Tagalene K, and Tagalsin C, exhibited cytotoxic effects at IC_50_ values ranging from 3.72 to 8.97 μM on various cancer cells, such as MDA‐MB‐453, MDA‐MB‐231, SK‐BR‐3, MT‐1, SW480, HeLa, PANC‐1, HCT‐8, Bel‐7402, BGC‐823, A549, and A2780 (Li et al., 2020; Ni et al., 2018; Yang et al., 2015; Zhang et al., 2018d). Furthermore, two investigations by different teams expressed that the silver nanoparticles prepared from leaf extracts of *Rhizophora apiculata* and Quinizarin derived from *Rhizospora mucronata*, displayed notable cytotoxic effects on MG‐63, HeLa, and MDA‐MB‐231 cancer cells (Sachithanandam et al., 2022a; Wen et al., 2020). Another study conducted by Tran et al. (2022) showed that 3‐*O*‐[*β*‐D‐xylopyranosyl‐(1 → 3)‐*β*‐D‐glucopyranosyl‐]‐oleanolic acid, isolated from *Lepisanthes rubiginosa*, showed cytotoxicity against KB, HepG2, SK‐LU‐1, and MCF‐7 cancer cells, and observable IC_50_ values were 9.57, 6.66, 6.97, and 18.32 μM, respectively (Tran et al., 2022). The plant *Aegiceras corniculatum* yielded several isolated compounds, namely Sakurasosaponin, Sakurasosaponin methyl ester, 5‐*O*‐butylembelin, 5‐*O*‐methylembelin, and 5‐*O*‐methyl‐rapanone, which demonstrated a remarkable cytotoxic action on MCF‐7, A549, B16F10, HCT116, HL‐60, HepG2, BGC‐823, and A2780 tumor cells, and the IC_50_ values of those representative compounds ranged from 2.21 to 10.6 μM (Li et al., 2020; Vinh et al., 2019). The extract from *Avicennia alba* and compound Dolichandrone B derived from *Dolichandrone spathacea* displayed cytotoxic action in KB, MCF‐7, and HeLa cancer cell lines (Eswaraiah, Peele, Krupanidhi, Kumar, & Venkateswarulu, 2020b; Nguyen et al., 2018). The results from another investigation manifested that the extract from *Excoecaria agallocha* and the preparation of silver nanoparticles had cytotoxic effects at given dosages of 25–100 μg/mL on cancer cells (MCF‐7, Capan‐1, Miapaca‐2, and A549) by upregulating the expression of p53 and p21 as well as suppressing the cell cycle (at G1 phase) (Bhuvaneswari et al., 2017; Patil et al., 2011, 2012). The extract of *Scyphiphora hydrophyllacea* has shown a notable impact on liver cancer cells (HepG2) by inducing DNA fragmentation and upregulating p53 expression, leading to a cytotoxic effect at 12.5–50 μg/mL dosage (Samarakoon et al., 2017). Several studies also demonstrated that the extract and isolated compounds, namely 1,5,6‐trihydroxy‐3‐methoxyxanthone, and Polygalatenoside E derived from *Lumnitzera racemosa*, showed cytotoxicity in HL‐60, MCF‐7, and HeLa cancer cells (Eswaraiah, Peele, Krupanidhi, Indira, et al., 2020a; Nguyen et al., 2015a). In addition, several studies have shown the cytotoxic effects of the extract and several compounds purified from *Xylocarpus moluccensis*, including Xylomolin J2, Thaixylogranins A–H, Godavarin L, and Godavarin N. These compounds have been found to have cytotoxic activity against certain cancer cells, such as MD‐MBA‐231, AGS, HT‐29, and MDA‐MB‐453, when treated at tested doses, and the IC_50_ range of those compounds was 2.1–61.1 μM (Liao et al., 2017; Uddin et al., 2011; Zhang, Li, Dai, et al., 2018a; Zhang, Shi, Liu, et al., 2018b). The compounds Patriscabratine, Tetracosane, and (7*S*, 8*R*)‐dehydroconiferyl alcohol, Sonnerphenolic C, as well as (7*S*, 8*R*)‐5‐methoxydehydroconiferyl alcohol, were obtained from the plant species *Acrostichum aureum* and *Sonneratia ovata* Backer, respectively. The compounds mentioned above exhibited a cytotoxic impact on cancer cells (AGS and MCF‐7), with IC_50_ range values 112.8 to higher than 250 μM (Nguyen, Pham, et al., 2015b; Uddin et al., 2012). Another study demonstrated that 3‐chlorodeoxylapachol derived from *Avicennia germinans* exhibited cytotoxic properties against KB cancer cells, and the extract obtained from *Avicennia officinalis* showed cytotoxic activity in mice at 200 and 400 mg/kg treatment dosages (Jones et al., 2005; Sumithra et al., 2011). Additionally, the compounds 17*β*‐neriifolin, 17*α*‐neriifolin, and Cerberin derived from *Cerbera odollam* showed cytotoxic effects on MCF‐7, T47D, SKOV3, CAOV3, KB, BC, and NCI‐H187 cancer cells with IC_50_ values ranging from 0.017 to 1.92 μg/mL (Laphookhieo et al., 2004; Syarifah et al., 2011). Likewise, it has been observed that 7,8‐dehydrocerberin, Deacetyltanghinin, and Tanghinin derived from *Cerbera manghas* exhibited cytotoxic effects on KB, BC, and NCI‐H187 cancer cells. The IC_50_ values for these compounds range from 0.0006 to 16.70 μg/mL (Cheenpracha et al., 2004). The compounds Hibiscone C and Syriacusin A were derived from *Hibiscus tiliaceus* and showed cytotoxic effects on HeLa cancer cells at a 30 μM concentration. Simultaneously, the extracts of *Phoenix paludosa* and *Suaeda maritima* exhibited cytotoxic properties and inhibited the proliferation of MCF‐7, MDA‐MB 231, SKBR‐3, MCF‐10A, ACHN, and HEK‐293 cancer cells. The IC_50_ values for extracts of *Phoenix paludosa* range from 26.99 to 159.7 μg/mL (Mohamed et al., 2022; Samarakoon et al., 2016; Μatsumoto et al., 2020). Moreover, silver nanoparticles, Everninic acid, Roccellic acid, and Harwickiic acid were isolated from the mangrove plants *Bruguiera cylindrica*, *Roccella montagnei*, and *Clerodendrum inerme*. These nanoparticles and phytochemicals exhibited cytotoxic properties and inhibited the proliferation of MCF‐7, DLD‐1, MDAMB‐231, and HCT116 cancer cells, with IC_50_ values ranging from 71.26 to more than 100 μg/mL (Ba Vinh et al., 2018; Bhuvaneswari et al., 2015; Mishra et al., 2017). The phytochemicals of Pinnatin, Derrischalcone, Tunicatachalcone, Obovatachalcone, Candidone, and (2*R*,3*R*)‐3‐hydroxy‐5‐methoxy‐2″,2″‐dimethylpyrano [7,8:5″,6″]‐flavanone from *Derris indica* showed cytotoxic effects on KKU‐100, HepG2, M156, and HepG2 cancer cells. The inhibition of cell growth was shown, with IC_50_ values ranging from 0.59 to 9.0 μg/mL (Decharchoochart et al., 2014; Saraphon et al., 2017). Mangrove plants and their bioactive phytochemicals acting against different types of cancer are presented in Table 2. Figure 3 illustrates the cytotoxic effect mechanism of mangrove plants and their active phytochemicals.

**TABLE 2:** Mangrove plants and their bioactive phytochemicals acting against cancer.

Plants (botanical name)	Extract/Compound/Nanoparticles	Type of cancer	Test system/Model/Cell lines	Tested dose	IC_50_ (Exposure time)	Anticancer effects and mechanisms	References
*Sonneratia paracaseolaris*	Paracaseolin D	–	A549, in vitro	–	1.89 μM	↑Cytotoxicity	Gong et al. (2017)
*Nypa fruticans*	Polyisoprenoid	Colon cancer	WiDr and 3T3, in vitro	–	180.2 and 397.7 μg/mL	↓Bcl‐2, ↓Cyclin D1, ↓cell cycle (G0–G1 phase), ↑apoptosis, ↓proliferation, ↑cytotoxicity	Sari et al. (2018)
*Avicennia marina*	Leaf extract	Breast cancer	MDA‐MB‐231, AU565, BT483, and HepG2, in vitro	40–200 μg/mL	–	↑Apoptosis, ↓migration, ↓proliferation, ↓colony formation, ↓ PARP, ↑caspase‐3 ↓MMP2, ↓MMP9, ↓cyclin B, ↓vimentin, and snail protein expression	Huang et al. (2016)
	Silver nanoparticles	Lung cancer	A549, in vitro	10–80 μg/mL	–	↑ROS generation, ↑mitochondrial damage	Tian et al. (2020)
	Avicennone A, Stenocarpoquinone B, Avicequinone C, Avicennone D, Avicennone E, Avicenol A, and Avicenol C	–	L‐929 and K562, HeLa, in vitro	–	0.80–13.10 μM	↓Proliferation, ↑cytotoxicity	Han et al. (2007)
	Luteolin 7‐*O*‐methylether 3′‐*O*‐*β*‐D‐glucoside	–	BT‐20, in vitro	–	16 μ/mL (ED_50_)	↑Cytotoxicity	Sharaf et al. (2000)
	Extract	–	HL‐60, in vitro	–	280 μg/mL (72 h)	↑Cytotoxicity	Karami et al. (2012)
	Extract	–	MCF‐7, in vitro	100 and 200 μg/mL	–	↓Mitochondrial membrane potential, ↑ROS generation, ↑apoptosis, ↑autophagy	Esau et al. (2015)
	Extract	–	HepG2, in vitro	25–400 μg/mL	–	↑Cytotoxicity	Sohaib et al. (2022)
	Lupeol	–	MCF‐7 and Hep3B, in vitro	50 μM	–	↓Bcl‐2, ↑apoptosis, ↑caspase‐3	Eldohaji et al. (2021)

	Saponin	–	GSC‐3 and GSC‐18, in vitro	–	12.21 and 5.53 μg/mL	↓Proliferation	Yang et al. (2018)
Silver nanoparticles	Lung cancer	A549, in vitro	10–100 μg/mL	50 μg/mL	↓Mitochondrial membrane potential, ↑ROS generation, ↑apoptosis, ↓Cyclin‐D1, ↑p21, ↑p53, ↓Bcl‐2, ↑Bax, ↑caspase‐3, ‐8, and ‐9, ↓cell cycle	Varunkumar et al. (2020)

	Extract and Luteolin	–	MDA‐MB‐231, in vitro	15–500 μg/mL	250 and 28 μg/mL	↑DNA fragmentation, ↑p53, ↓Bcl‐2, ↑apoptosis, ↓proliferation	Momtazi‐Borojeni et al. (2013)
Naphtho[1,2‐b] furan‐4,5‐dione	Lung cancer	H1299, H1437, and A549, in vitro	1–10 μM	1.66–6.29 μM	↓Proliferation, ↑ROS generation, ↑DNA damage, ↑apoptosis, ↓Bcl‐2, ↑Bax, ↓Topoisomerase II, ↓NF‐κB	Chien et al. (2019)
*Xylocarpus granatum*	Gedunin	Ovarian cancer	PA‐1 and OVCAR‐3, in vitro	5–30 μM	8.1 and 18 μM (48 h)	↑ROS generation, ↑DNA damage, ↓cell cycle (G2/M phase), ↑cytochrome C, ↑caspase‐9 and‐3 activation, ↓PARP, ↑p21, ↑p53, ↓Bcl‐2, ↑Bax, ↑apoptosis, ↓Heat shock protein 90 (HSP90), ↓migration, ↓proliferation	Sahai et al. (2020)
	Extract	–	HT‐29, in vitro	–	23.12 ppm	↓Proliferation	Darmadi et al. (2021)
	Granaxylocarpin A and Granaxylocarpin B	–	P‐388, in vitro	–	9.3 and 4.9 μM	↑Cytotoxicity	Yin et al. (2007)
	Xylogranatin A, Xylogranatin B, Xylogranatin C, and Xylogranatin D	–	P‐388 and A‐549, in vitro	–	6.3–15.7 μM	↑Cytotoxicity	Yin et al. (2006)
	Gedunin	Colon cancer	CaCo‐2, in vitro	–	16.83 μM	↑Cytotoxicity	Uddin et al. (2007)
	Xylogranatumine F	–	A549, in vitro	–	10 μM	↑Cytotoxicity	Zhou et al. (2014)
	Xylogranatin C, 7‐oxo‐7‐deacetoxygenudin, and 7‐deacetylgenudin	–	CHAGO and Hep‐G2, in vitro	–	9.16–16.17 μM	↑Cytotoxicity	Pudhom et al. (2009)
	Xylomexicanin A	–	KT, in vitro	–	4.59 μM	↓Proliferation	Shen et al. (2009)
	Xylogranin B	–	SW480 and HCT116, in vitro	–	48.9 nM	↓Wnt/ β‐catenin signaling, ↓c‐myc and PPARδ, ↓TCF/β‐catenin transcription, ↑cytotoxicity	Toume et al. (2013)
	Xylomexicanin F	–	A549 and RERF, in vitro	–	18.83 and 15.83 μM	↓Proliferation	Wu, Qing, et al. (2014b)
*Ceriops tagal*	Tagalene I, Tagalon C, and Tagalon D	Breast cancer	MDA‐MB‐453, MDA‐MB‐231, SK‐BR‐3, and MT‐1, in vitro	–	3.75–8.97 μM	↑Cytotoxicity	Zhang, Li, Shen, and Wu (2018d)
	Gallic acid and Quercetin	–	HeLa, MDA‐MB231, in vitro	–	4.18, 80.04 μg/mL (24 h) and 99.91, 18.29 μg/mL (24 h)	↓Proliferation	Sachithanandam et al. (2022b)

	Tagalene K	–	SW480, HeLa, and PANC‐1, in vitro	–	27.7, 22.2, and 17.6 μM	↑Cytotoxicity	Ni et al. (2018)
	Tagalsin C	–	HCT‐8, Bel‐7402, BGC‐823, A549, and A2780, in vitro	–	3.72–8.85 μM	↑Cytotoxicity	Yang et al. (2015)
	Tagalsin C	–	Jurkat, in vitro	–	–	↑ROS generation, ↑DNA damage, ↓cell cycle (S–G2 phase), ↑apoptosis, ↑ATM/ATR and ↓Chk1/Chk2 check point pathway	Neumann et al. (2015)
	Quercetin and gold nanoparticles	–	A549 and HeLa, in vitro	–	79.9 and 73 μg/mL	↓Proliferation	Parthiban et al. (2023)
	Botulin and 3‐epi‐betulinic acid acetate	–	H‐7402 and Hela, in vitro	–	14.42, 9.97 μg/mL and 11.84,11.32 μg/mL	↓Proliferation	He et al. (2007)
Tagalide A	Breast cancer	MD‐MBA‐453 and MDA‐MB‐231, in vitro	–	1.73 and 8.12 μM	↓Phosphorylation of JAK2 and STAT3, ↑ROS generation, ↑apoptosis, ↓cell cycle (G2/M phase)	Zhang et al. (2018c)
*Rhizospora mucronata*	Quinizarin	–	HeLa and MDA‐MB‐231, in vitro	0.5–100 μg/mL	4.60 and 3.89 μg/mL	↑Cytotoxicity	Sachithanandam, Lalitha, et al. (2022a)
	Silver nanoparticles	–	MCF‐7, in vitro	5–75 μg/mL	–	↑Apoptosis, ↓proliferation	Rajivgandhi et al. (2022)
*Rhizophora apiculata*	Extract	Lung cancer	BALB/c mice, in vivo	10 mg/kg	–	↓Solid tumor development, ↓GSH, ↓GGT, ↓NO, ↓metastasis	Prabhu and Guruvayoorappan (2012, 2013)
	Silver nanoparticles, leaf extract	Bone cancer	MG‐63, in vitro	–	–	↑Cytotoxicity	Wen et al. (2020)
	1,2‐diazole	Lung cancer	A549, in vitro	5–500 μM	75 μM	↓Cell cycle (G1–S phase), ↑apoptosis, ↓EGFR tyrosine kinase, ↓mitochondrial membrane potential, ↓CDK‐2, ↓Bcl‐2, ↑Bax	Vinod Prabhu et al. (2018)
*Lepisanthes rubiginosa*	3‐*O*‐[*β*‐D‐xylopyranosyl‐(1 → 3)‐*β*‐D‐glucopyranosyl‐]‐oleanolic acid	–	KB, HepG2, SK‐LU‐1, and MCF‐7, in vitro	–	9.57, 6.66, 6.97, and 18.32 μM	↑Cytotoxicity	Tran et al. (2022)
*Aegiceras corniculatum*	Sakurasosaponin and Sakurasosaponin methyl ester	–	MCF‐7, A549, B16F10, and HCT116, in vitro	–	2.21–9.85 μM	↑Cytotoxicity	Vinh et al. (2019)
	5‐*O*‐butylembelin, 5‐*O*‐methylembelin, and 5‐*O*‐methyl‐rapanone	–	HL‐60, HepG2, BGC‐823, and A2780, in vitro	–	7.6–10.6 μM	↑Cytotoxicity	Li et al. (2020)
	Extract	Colorectal cancer	HT‐29, SW480, in vitro	25–100 μg/mL	34.01–61.28 μg/mL	↑Apoptosis, ↓Bcl‐2, ↑Bax, ↑caspase‐3, ‐8, and ‐9 ↓proliferation, ↓cell cycle, ↓Cyclin D3, ↓Cyclin D1, ↓CDK2, ↓CDK4, and ↓CDK6, ↑p21 and p27, ↑Foxo1, and Foxo3a	Luo et al. (2019)
*Derris trifoliata*	Silver nanoparticles and seed extract	–	A549, in vitro	6.25–100 μg/mL	86.23 and greater than 100 μg/mL	↓Proliferation	Cyril et al. (2019)
*Dolichandrone spathacea*	Dolichandrone B	–	KB, in vitro	–	18.77 μM	↑Cytotoxicity	Nguyen et al. (2018)
*Avicennia alba* Blume	Polyisoprenoids	Colorectal cancer	WiDr, in vitro	–	173.78 μg/mL	↓Cell cycle (G0–G1 phase), ↓COX‐2 expression, ↑apoptosis	Illian et al. (2019)
	Extract	–	MCF‐7 and HeLa, in vitro	20–100 μg/mL	57.02 and 44.30 μg/mL	↑Cytotoxicity	Eswaraiah, Peele, Krupanidhi, Kumar, and Venkateswarulu (2020b)
*Excoecaria agallocha* L.	Silver nanoparticles	Breast cancer	MCF‐7, in vitro	–	–	↑Cytotoxicity	Bhuvaneswari et al. (2017)
	Extract	–	Capan‐1, Miapaca‐2, and A549, in vitro	25–100 μg/mL	4 and 7 μg/mL	↑Cytotoxicity, ↓cell cycle (G1 phase), ↑apoptosis, ↑p21, ↑p53, ↓Bcl‐2, ↑Bax	Patil et al. (2012), Patil et al. (2011)
*Scyphiphora hydrophyllacea*	Extract	Liver cancer	HepG2, in vitro	12.5–50 μg/mL	–	↑Cytotoxicity, ↑DNA fragmentation, ↑p53, ↑Bax, ↑apoptosis, ↑caspases‐3/9	Samarakoon et al. (2017)
*Lumnitzera racemosa*	1,5,6‐trihydroxy‐3‐methoxyxanthone and polygalatenoside E	–	HL‐60, in vitro	–	0.15 and 0.60 μM	↑Cytotoxicity	Nguyen, Bui, et al. (2015a)
	Extract	–	MCF 7 and HeLa, in vitro	20–100 μg/mL	46.098 and 59.497 μg/mL	↑Cytotoxicity	Eswaraiah et al. (2020a)
*Xylocarpus moluccensis*	Xylomolin J2	–	MD‐MBA‐231, in vitro	–	37.7 μM	↑Cytotoxicity	Zhang et al. (2018d)
	Extract	–	AGS, HT‐29, and MDA‐MB‐435, in vitro	–	0.2–2.3 μg/mL	↑Cytotoxicity	Uddin et al. (2011)
	Thaixylogranins A–H	–	MDA‐MB‐231, in vitro	–	38.5–61.1 μM	↑Cytotoxicity	Liao et al. (2017)
	Godavarin L and Godavarin N	Breast cancer	MDA‐MB‐453, in vitro	–	2.1 and 9.0 μM	↑Cytotoxicity	Zhang, Shi, Liu, et al. (2018b)
	Extract	Liver cancer	HepG2, in vitro	–	25.12 μg/mL	↑DNA fragmentation, ↑apoptosis, ↑caspases‐8 and ‐3/7	Chaudhry et al. (2021)
*Acrostichum aureum* L.	Patriscabratine and Tetracosane	–	AGS, in vitro	–	133.6 and greater than 250 μM	↑Cytotoxicity, ↑apoptosis	Uddin et al. (2012)
*Chaetomorpha brachygona*	Extract	Cervical cancer	SiHa, in vitro	–	–	↑Autophagy, ↑AMPKα ↑Beclin‐1, ↑S6, ↑p62, ↑LC3BII, ↓proliferation, ↓mitochondrial membrane potential, ↑ROS generation, ↑apoptosis	Majumder et al. (2020)
*Sonneratia ovata* Backer	(7*S*,8*R*)‐dehydroconiferyl alcohol, sonnerphenolic C, (7*S*,8*R*)‐5‐methoxydehydroconiferyl alcohol	–	MCF‐7, in vitro	–	146.9, 112.8, and 114.5 μM	↑Cytotoxicity	Nguyen, Pham, et al. (2015b)
*Acanthus ilicifolius*	Extract	–	Rat, in vivo	250 mg/kg	–	↓Lipid peroxidation and MDA, ↓aberrant crypt foci, ↓Bcl‐2, ↑Bax, ↑p53, ↓proliferating nuclear cell antigen, ↑apoptosis	Almagrami et al. (2014)
*Avicennia germinans*	3‐chlorodeoxylapachol	–	KB, in vitro	–	–	↑Cytotoxicity	Jones et al. (2005)
*Avicennia officinalis*	Extract	–	Mice, in vivo	200 and 400 mg/kg	–	↑Cytotoxicity	Sumithra et al. (2011)
*Heritiera fomes* Buch.	Extract	–	B16, in vitro	–	75 μg/mL	↓Proliferation	Patra and Thatoi (2013)
*Ceriops decandra*	Extract	–	Male hamsters, in vivo	5 mg/kg	–	↓Buccal pouch carcinogenesis	Boopathy et al. (2011)
*Cerbera odollam*	17*β*‐neriifolin	–	MCF‐7, T47D, SKOV3, and CAOV3, in vitro	–	17–32 nM	↑Cytotoxicity	Syarifah et al. (2011)
	17*α*‐neriifolin, 17*β*‐neriifolin, and cerberin	–	KB, BC, and NCI‐H187, in vitro	–	0.017–1.92 μg/mL	↑Cytotoxicity	Laphookhieo et al. (2004)
*Cerbera manghas*	14‐hydroxy‐3*β*‐(3‐*O*‐methyl‐6‐deoxy‐*α*‐L‐rhamnosyl)‐11*α*,12*α*‐epoxy‐(5*β*,14*β*,17*βH*)‐card‐20(22)‐enolide 14‐hydroxy‐3*β*‐(3‐*O*‐methyl‐6‐deoxy‐*α*‐L‐glucopyranosyl)‐11*α*,12*α*‐epoxy‐(5*β*,14*β*,17*βH*)‐card‐20(22)‐enolide and 17*β*‐neriifolin	–	Col2, in vitro	–	0.015–0.100 μg/mL	↓Proliferation	Chang et al. (2000)
	7,8‐Dehydrocerberin, deacetyltanghinin, and tanghinin	–	KB, BC, and NCI‐H187, in vitro	–	0.0006–16.70 μg/mL	↑Cytotoxicity	Cheenpracha et al. (2004)
	Tanghinigenin	–	HL‐60, in vitro	–	2.15 μM	↑Caspase‐3, ‐8, and ‐9, ↑Fas and FasL, ↑apoptosis, ↓proliferation	Wang et al. (2010)
	Neriifolin	Hepatocellular carcinoma	HepG2, in vitro	0.05–8 μg/mL	0.15 μg/mL	↑Caspase‐3, ‐8, and ‐9, ↑Fas and FasL, ↑apoptosis, ↓proliferation, ↓cell cycle (S–G2/M phase)	Zhao et al. (2011)
*Hibiscus tiliaceus*	Hibisceusones A–C	Breast cancer	MDA‐MB‐231, in vitro	–	–	↑Apoptosis, ↓proliferation, ↓PI3Kα pathway	Chen et al. (2022)
	Hibiscone C and Syriacusin A	–	HeLa, in vitro	30 μM	–	↑Cytotoxicity	Μatsumoto et al. (2020)
*Phoenix paludosa* Roxb.	Extract	–	MCF‐7, MDA‐MB‐231, SKBR‐3, MCF‐10A, ACHN, HEK‐293, in vitro	–	26.99–159.7 μg/mL	↓Proliferation, ↑cytotoxicity	Samarakoon et al. (2016)
*Suaeda maritima*	Extract	–	–	–	–	↑Cytotoxicity	Mohamed et al. (2022)
*Bruguiera cylindrica*	Silver nanoparticles	–	MCF‐7, in vitro	50, 100 μg/mL	100 μg/mL	↑Cytotoxicity	Bhuvaneswari et al. (2015)
*Roccella montagnei*	Everninic acid and Roccellic acid	–	DLD‐1, MCF‐7, and MDA‐MB‐231, in vitro	6.25–100 μg/mL	71.26 − greater than 100 μg/mL	↑Cytotoxicity, ↓proliferation	Mishra et al. (2017)
*Clerodendrum inerme*	Harwickiic acid	–	HCT116, in vitro	–	75.41 μM	↑Cytotoxicity	Ba Vinh et al. (2018)
*Thespesia populnea*	3‐*O*‐methylthespesilactam	–	A2058, in vitro	10–40 μM	–	↑Apoptosis, ↓cell cycle (S phase), ↓phosphorylation of JAK1, JAK2, TYK2, and STAT3, ↓proliferation, ↓Bcl‐2, ↓PARP, ↓Mcl‐1	Li et al. (2013)
*Caulerpa scalpelliformis*	Silver nanoparticles	–	MCF‐7, in vitro	10–250 μg/mL	40 μg/mL	↓Bcl‐2, ↑Bax, ↑caspase‐3 and ‐9 ↓proliferation, ↑apoptosis	Manikandan et al. (2019)
*Derris indica*	Pinnatin	–	KKU‐100 and HepG2, in vitro	–	6.0 and 9.0 μg/mL	↑Cytotoxicity	Saraphon et al. (2017)
	Derrischalcone, Tunicatachalcone, Obovatachalcone, Candidone, and (2*R*,3*R*)‐3‐hydroxy‐5‐methoxy‐2″,2″‐dimethylpyrano [7,8:5″,6″]‐flavanone	–	M156 and HepG2, in vitro	–	0.59–7.80 and 2.60–11.20 μg/mL	↑Cytotoxicity	Decharchoochart et al. (2014)

*Note*: Arrows (↑ and ↓) show an increase and decrease in the obtained variables.

Abbreviations: AMPK, AMP‐activated protein kinase; ATM, Ataxia telangiectasia *mutated*; Bax, Bcl‐2‐associated X protein; Bcl‐2, B‐cell lymphoma 2; CDK 2,4,6, cyclin‐dependent kinase 2,4,6; COX‐2, cyclooxygenase 2; EGFR, epidermal growth factor receptor; Foxo1, forkhead box protein O1; Foxo3a, forkhead box O3; GGT, gamma‐glutamyl transferase; GSH, glutathione; JAK1, Janus kinase 1; JAK2, Janus kinase 2; JAK2, Janus kinase 2; LC3BII, microtubule‐associated protein light chain 3; Mcl‐1, myeloid cell leukemia sequence 1; MDA, malondialdehyde; MMP2, matrix metalloproteinase‐2; MMP9, matrix metalloproteinase‐9; NF‐κB, nuclear factor‐kappa‐light‐chain‐enhancer of activated B cells; NO, nitric oxide; p21, tumor protein p21; p53, tumor protein p53; PARP, poly (ADP‐ribose) polymerase; PI3K, phosphoinositide 3‐kinase; ROS, reactive oxygen species; S6, ribosomal protein S6; STAT3, signal transducer and activator of transcription 3; TYK2, tyrosine‐kinase 2.

#### 3.3.3 Genotoxicity

The term genotoxicity pertains to the capacity of a chemical to induce harm to the genetic material of an organism, especially targeting its DNA (Ren et al., 2017). Such damage can induce mutations, chromosomal abnormalities, or other alterations in the genetic material, hence giving rise to a range of detrimental health consequences, such as cancer development (Basu, 2018; Torgovnick & Schumacher, 2015). Recent studies show the efficacy of genotoxic drugs in inducing DNA damage. Additionally, these treatments can impede cell cycle checkpoints, leading to cell cycle arrest and/or cell death (Singh et al., 2018; Swift & Golsteyn, 2014).

Based on a study report, mangrove plants and their derived phytochemicals have the potential to initiate the process of DNA damage. The chemical Naphtho[1,2‐b] furan‐4,5‐dione, extracted from *Avicennia marina*, has been shown to have genotoxic effects on lung cancer cells. This was achieved through its ability to enhance DNA damage while simultaneously reducing the activity of Topoisomerase II (Chien et al., 2019). Similarly, Gedunin, derived from *Xylocarpus granatum*, has been shown to enhance DNA damage by reducing Poly (ADP‐ribose) polymerase (PARP) activity and increasing the expression of tumor protein p21 (p21) and tumor protein p53 (p53). Additionally, it induces cell cycle arrest in the G2/M phase (Sahai et al., 2020). The study by Neumann et al. (2015) shows that Tagalsin C derived from *Ceriops tagal* induced an increase in DNA damage and cell cycle arrest in the S–G2 phase. The genotoxic effect was mediated by activating the Ataxia telangiectasia *mutated*/acidosis tubulare renale–checkpoint kinases 1 and 2 (ATM/ATR–Chk1/Chk2) pathway (Neumann et al., 2015). Figure 3 shows the genotoxic effect mechanism of mangrove plants and their active phytochemicals.

#### 3.3.4 Cell cycle arrest

The cell cycle embodies a sequence of intricately coordinated occurrences that enable cellular expansion and propagation (Loftus et al., 2022). Cancer embodies an aberration in this cycle, where cells either excessively produce cyclins or fail to express cyclin‐dependent kinase (CDK) inhibitors, resulting in uncontrolled cellular proliferation (Schwartz & Shah, 2005). Since the cell cycle functions as a safeguard against DNA damage (Ahmed et al., 2023), halting the cell cycle is a protective strategy, allowing malignant cells to mend their DNA impairment (Hohensinner et al., 2014). Interfering with the cell cycle curtails the unchecked proliferation of tumor cells and initiates the apoptotic effect (Tang et al., 2010). Thus, targeting cell cycle arrests in cancer therapy is a promising strategy (Huang & Zhou, 2020). The advancement of cancer cells into tumors and their dissemination to other body regions are thwarted by impeding the cell cycle (Emami Nejad et al., 2021).

Studies have demonstrated that mangrove plants and their bioactive chemicals exert an anticancer effect by hindering different cell cycle phases. For instance, Polyisoprenoids from *Nypa fruticans* leaves have anticancer activity by diminishing the manifestation of Cyclin D1, facilitating the arrest of the cell cycle in the G0–G1 phase in colon cancer cells (Sari et al., 2018). At the same time, the silver nanoparticles synthesized from Avicennia marina exhibited anticancer activity against lung cancer cells by inhibiting the cell cycle via downregulation of Cyclin D1 and upregulation of p21 and p53 transcription factor protein expression (Varunkumar et al., 2020). Similarly, Gedunin from *Xylocarpus granatum* exhibited a DNA damage response in ovarian cancer cells and upregulated p21 and p53 protein expression, inhibiting the cell cycle and decreasing cell proliferation (Sahai et al., 2020). Furthermore, Tagalsin C from *Ceriops tagal* initiated ROS‐mediated DNA damage. This event leads to blockage of cell cycle progression at the S–G2 phase via activation of the ATM/ATR–Chk1/Chk2 checkpoint pathway (Neumann et al., 2015). In the study of Tagalide A from *Ceriops tagal*, blockage of cell cycle progression at the G2/M phase was also reported in breast cancer cells (Zhang, Yang, Liu, et al., 2018c). In 2018, Vinod Prabhu and colleagues found that 1,2‐diazole (Pyrazole) extracted from *Rhizophora apiculata* significantly suppresses the expression of cyclin‐dependent kinase 2 (CDK2), resulting in cell cycle arrest at the G1 phase and in the G1–S phase transition (Vinod Prabhu et al., 2018). According to Luo et al. (2019), the 95% ethanol extract of *Aegiceras corniculatum* leaves suppressed the cell cycle in colorectal cancer via downregulation of cell cycle regulator molecules like Cyclin D3, Cyclin D1, and blockage of cyclin‐dependent kinase 2 (CDK2), cyclin‐dependent kinase 4 (CDK4), and cyclin‐dependent kinase 6 (CDK6), as well as upregulation of tumor protein p21 (p21) and tumor protein p27 (p27) protein expression (Luo et al., 2019).

**FIGURE 4:**
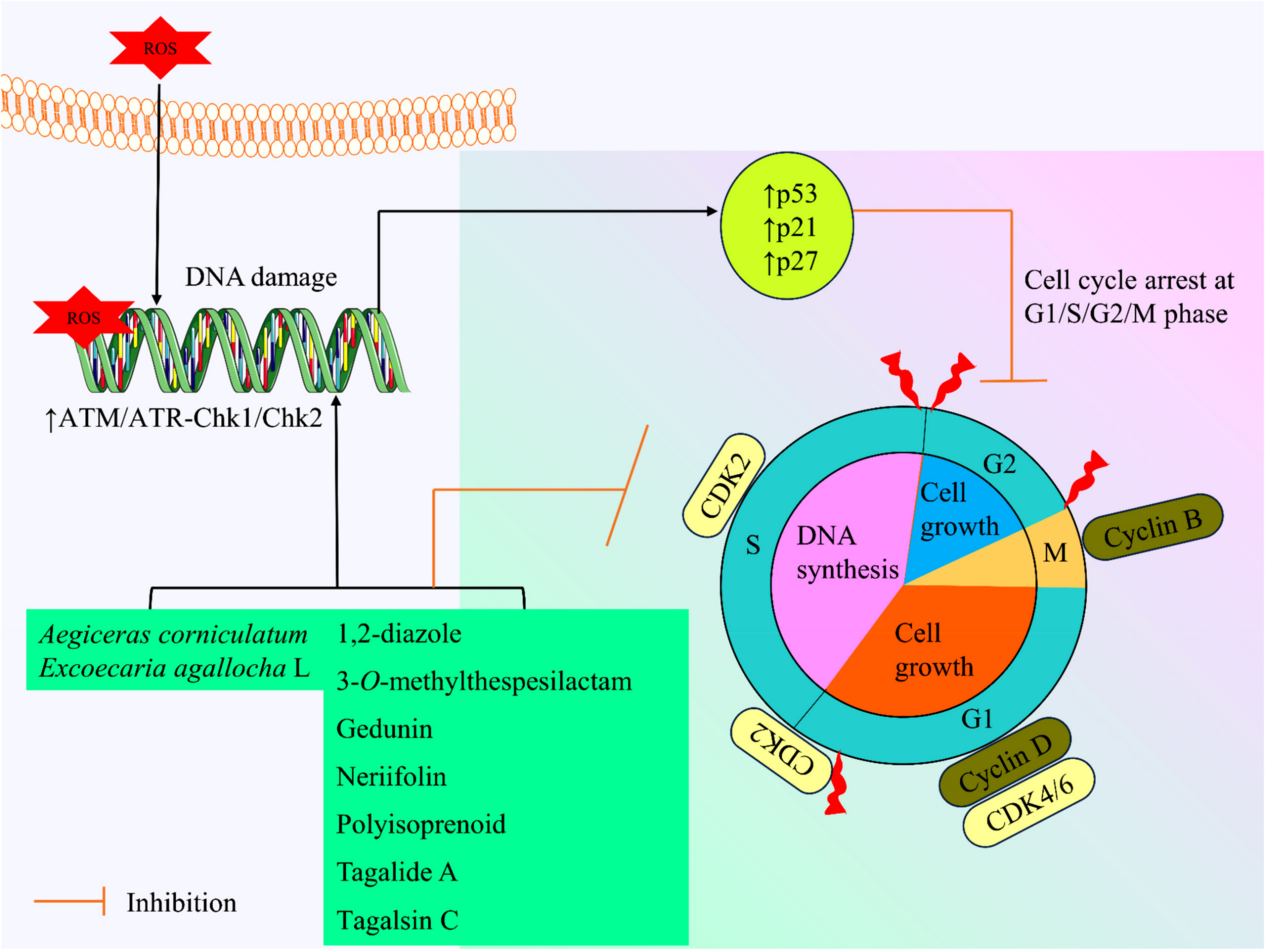
The possible cell cycle arrest mechanisms of mangrove plants and their derived compounds (ATM, Ataxia telangiectasia *mutated*; p21, tumor protein p21; p27, tumor protein p27; p53, tumor protein p53; ROS, reactive oxygen species).

Moreover, Polyisoprenoids isolated from *Avicennia alba* have significantly suppressed the cell cycle at the G0–G1 phase in the colorectal cancer cell (Illian et al., 2019). The ethanolic extract of *Excoecaria agallocha* also significantly blocked cell cycle progression via activation of p21 and p53 expression (Patil et al., 2011, 2012). Neriifolin from *Cerbera manghas* has shown cell cycle arrest activity in hepatocellular carcinoma cells at the S–G2/M phase (Zhao et al., 2011). In a study on 3‐*O*‐methylthespesilactam from *Thespesia populnea*, it can arrest the cell cycle and suppress cancer cell proliferation (Li et al., 2013). The possible cell cycle arrest mechanisms of mangrove plants and their derived compounds are illustrated in Figure 4.

#### 3.3.5 Apoptotic effect

Apoptosis, or programmed cell death, is orchestrated by intricate molecular mechanisms (Nakka et al., 2008). Two main intrinsic and extrinsic pathways converge to activate caspases, the executioner proteins that dismantle cells (D'arcy, 2019; Wu, Che, et al., 2014a). In the intrinsic pathway, cellular stress prompts Bcl‐2 family proteins to permeabilize mitochondrial membranes, releasing cytochrome C. This triggers caspase activation via the apoptosome (Kalkavan & Green, 2018). The extrinsic pathway starts with death receptors binding to ligands, forming complexes that activate caspase‐8, initiating cell dismantling (Tait & Green, 2010). These pathways intertwine and culminate in caspase‐3 activation, resulting in cellular changes such as DNA fragmentation and membrane blebbing (Orning & Lien, 2021).

Numerous studies have provided evidence that mangrove plants and their derived compounds could elicit an anticancer impact by activating the apoptotic pathway in preclinical studies. Polyisoprenoids derived from *Nypa fruticans* leaves exhibit potential anticancer properties by downregulating the expression of Bcl‐2, hence promoting apoptosis in cancer cells (Sari et al., 2018). The extract of the *Avicennia marina* plant, together with its derived chemical, induces apoptosis in different types of cancer cells by reducing the expression of PARP, decreasing mitochondrial membrane potential, increasing ROS generation, and enhancing caspase‐3 activity (Esau et al., 2015; Huang et al., 2016). These studies provided evidence that the compounds Lupeol, Luteolin, and silver nanoparticles can trigger apoptosis. This effect was achieved through the stimulation of ROS production, fragmentation of DNA, and the upregulation of several proteins, including p21, p53, and Bcl‐2‐associated X protein (Bax), as well as the activation of caspase‐3, ‐8, and ‐9 enzymes. The attenuation in mitochondrial membrane potential and the downregulation of Bcl‐2 activity were also observed in cancer cells (Eldohaji et al., 2021; Momtazi‐Borojeni et al., 2013; Varunkumar et al., 2020). The study conducted by Chien et al. (2019) presented evidence showcasing the capacity of Naphtho[1,2‐b] furan‐4,5‐dione to promote apoptosis through the augmentation of ROS production, DNA damage, and the upregulation of Bax expression, as well as a reducetion in Bcl‐2 activity (Chien et al., 2019). In this connection, the compound Gedunin, derived from *Xylocarpus granatum*, has been found to enhance the production of ROS, induce DNA damage, promote the secretion of cytochrome C, and activate caspase‐3/9 in cancer cells. Additionally, it upregulates the expression of p21, p53, and Bax. Furthermore, the diminishment in PARP and Bcl‐2 expression leads to the initiation of apoptosis (Sahai et al., 2020). Tagalsin C and Tagalide A derived from *Ceriops tagal* could cause apoptosis by increasing the production of ROS and causing DNA damage. Additionally, these compounds downregulate the phosphorylation of the Janus kinase 2 (JAK2) and signal transducer and activator of transcription 3 (STAT3) pathways (Neumann et al., 2015; Zhang et al., 2018c). According to Vinod Prabhu et al. (2018) observed that the compound 1,2‐diazole, derived from *Rhizophora apiculata*, exhibited the ability to attenuate the mitochondrial membrane potential and downregulate the expression of Bcl‐2 while simultaneously upregulating the expression of Bax. These molecular changes ultimately led to the induction of apoptosis. Additionally, the chemical exhibits inhibitory effects on the epidermal growth factor receptor (EGFR) tyrosine kinase (Vinod Prabhu et al., 2018). The *Aegiceras corniculatum* extract induces apoptosis in a cancer cell via activating the forkhead box protein O (Foxo) signaling pathway, leading to an increase in the activation of p21, p27, Bax, caspase‐3, ‐8, and ‐9, and a decrease in the expression of the antiapoptotic protein Bcl‐2 (Luo et al., 2019). The Polyisoprenoid compounds derived from *Avicennia alba* exhibited an apoptotic effect through the inhibition of cyclooxygenase 2 (COX‐2) expression (Illian et al., 2019). The extract of *Excoecaria agallocha* exhibits upregulation of p21, p53, and Bax proteins while downregulating the Bcl‐2 protein. This modulation of protein expression promotes apoptosis in cancer cells (Patil et al., 2011, 2012). Another investigation by Samarakoon et al. (2017) documented the apoptotic properties of *Scyphiphora hydrophyllacea* extract by activating DNA fragmentation, p53, Bax, and caspase‐3/9 activity (Samarakoon et al., 2017). In addition, the extract of *Chaetomorpha brachygona* induces an attenuation in mitochondrial membrane potential and an elevation in the production of ROS (Majumder et al., 2020). The apoptotic effects of mangrove plant extracts of *Xylocarpus moluccensis* and *Acanthus ilicifolius*, as well as the isolated compounds Patriscabratine and Tetracosane from *Acrostichum aureum* enhance the process of apoptosis in cancer cells. This was achieved through the upregulation of DNA fragmentation and the activation of many proteins associated in apoptosis, such as p53, Bax, and caspase‐8, ‐3, and ‐7 (Almagrami et al., 2014; Chaudhry et al., 2021; Uddin et al., 2012). The compounds Tanghinigenin and Neriifolin derived from *Cerbera manghas* induce apoptosis in cancer cells by activating death receptors (Fas and Fas ligand (FasL)) and upregulating apoptotic proteins including caspase‐3, ‐8, and ‐9 (Wang et al., 2010; Zhao et al., 2011). Recently, it was documented that Hibisceusones A–C derived from *Hibiscus tiliaceus* and the silver nanoparticles from *Rhizophora mucronata* could induce apoptosis in cancer cells (Chen et al., 2022; Rajivgandhi et al., 2022). The compound 3‐*O*‐methylthespesilactam, isolated from *Thespesia populnea*, and silver nanoparticles made from *Caulerpa scalpelliformis* were found to downregulate the phosphorylation of Janus kinase 1 (JAK1), JAK2, tyrosine‐kinase 2 (TYK2), and STAT3 proteins, as well as Bcl‐2, PARP, and myeloid cell leukemia sequence 1 (Mcl‐1) protein expression. Additionally, both preparations upregulated the apoptotic proteins Bax, caspase‐3 and ‐9, leading to apoptosis in cancer cells (Li et al., 2013; Manikandan et al., 2019).

#### 3.3.6 Antiproliferative effect

Normal cells have strictly controlled cell proliferation, while cancer cells exhibit excessive cell proliferation due to growth suppressor evasion and proliferative signals (Hanahan & Weinberg, 2011). Antiproliferative drugs inhibit the growth of cancer cells by controlling gene expression, which is started via signal transduction pathways (Tin et al., 2012). By controlling the signaling of nuclear receptors, chromatin remodelers, growth factor receptor tyrosine kinases, serine/threonine kinases, transcription factors, and cell cycle effectors, antiproliferative drugs stop cancer cells from multiplying (Abbastabar et al., 2018; Asmana Ningrum, 2014). However, it is essential to note that antiproliferative drugs possess inherent potential for adverse effects, including gastrointestinal complications, liver and renal dysfunction, muscle and joint pain, hair loss, and fatigue (Anand et al., 2023). Consequently, there is a pressing need to develop improved antiproliferative treatments that exhibit minimal side effects.

Multiple studies proved that, owing to their antiproliferative properties, mangrove plants and their active chemicals may be a great choice for studies looking into potential anticancer effects. The substance Polyisoprenoids was extracted from *Nypa fruticans* and has inhibitory effects on the proliferation of colon cancer cells. This anticancer mechanism was achieved by hindering the cell cycle regulatory protein Cyclin D1, leading to a halt in the progression of the cell cycle, specifically in the G0–G1 phase (Sari et al., 2018). The *Avicennia marina* mangrove plant has various chemicals, including Avicennone A, Stenocarpoquinone B, Avicequinone C, Avicennone D, Avicennone E, Avicenol A/C, Saponin, Luteolin, and Naphtho[1,2‐b] furan‐4,5‐dione. These chemicals have demonstrated the ability to suppress the proliferation of cancer cells by inducing DNA fragmentation, DNA damage, and elevated expression of the p53 protein. Additionally, they have shown inhibitory effects on PARP, cyclin B, Topoisomerase II, and nuclear factor kappa‐light‐chain‐enhancer of activated B cells (NF‐κB) protein activity (Chien et al., 2019; Han et al., 2007; Huang et al., 2016; Momtazi‐Borojeni et al., 2013; Yang et al., 2018). Similarly, the compound derived from the plant *Xylocarpus granatum*, known as Gedunin, has been shown to impede the growth of cancer cells. This effect was achieved by upregulating DNA damage markers, namely p21 and p53 protein expression, and suppressing PARP and heat shock protein 90 (HSP90). Additionally, Gedunin controlled the cell cycle during the G2/M phase (Sahai et al., 2020). Some studies demonstrated that the plant extract and the compounds Xylomexicanin A and Xylomexicanin F exhibited a reduction in cancer cell proliferation (Darmadi et al., 2021; Shen et al., 2009; Wu, Qing, et al., 2014b). The antiproliferative effects of Gallic acid, Quercetin, Botulin, 3‐epi‐betulinic acid acetate, and gold nanoparticles derived from *Ceriops tagal* have been shown to successfully inhibit the growth of several types of cancer cells (He et al., 2007; Parthiban et al., 2023; Sachithanandam et al., 2022b). Based on empirical investigations, the extract derived from *Aegiceras corniculatum* exhibited an upregulation in the expression of p21, P27, Forkhead box protein O1 (Foxo1), and Forkhead box protein O3 (Foxo3a) while concurrently downregulating the activity of Cyclin D3, Cyclin D1, CDK2, ‐4, and ‐6 proteins. This extract also demonstrated the ability to impede the cell cycle, effectively regulating the proliferation of cancer cells (Luo et al., 2019). *Derris trifoliata* silver nanoparticles and seed extract effectively inhibit the proliferation of cancer cells (Cyril et al., 2019). In a study conducted in 2020, Majumder et al. (2020) observed that the chloroform fraction derived from *Chaetomorpha brachygona* could induce autophagy in cervical cancer cells, leading to a reduction in cell growth. Similarly, the application of *Acanthus ilicifolius* extract can block cell growth via the upregulation of p53 expression and the induction of apoptosis (Almagrami et al., 2014). The extract derived from *Heritiera fomes* has significant efficacy in inhibiting the growth of cancer cells (Patra & Thatoi, 2013). The identified chemicals derived from *Cerbera manghas*, including 14‐hydroxy‐3*β*‐(3‐*O*‐methyl‐6‐deoxy‐*α*‐L‐rhamnosyl)‐11*α*,12*α*‐epoxy‐(5*β*,14*β*,17*βH*)‐card 20(22)‐enolide, 14‐hydroxy‐3*β*‐(3‐*O*‐methyl‐6‐deoxy‐*α*‐L‐glucopyranosyl)‐11*α*,12*α*‐epoxy‐(5*β*,14*β*,17*βH*)‐card‐20(22)‐enolide, 17*β*‐neriifolin, Tanghinigenin, and Neriifolin, exhibited enhanced death cell receptor activity. These compounds could control cancer cell apoptosis and stop the cell cycle at the G2/M phase, reducing cell proliferation (Chang et al., 2000; Wang et al., 2010; Zhao et al., 2011). In contrast, silver nanoparticles derived from *Rhizophora mucronata* have been shown to effectively regulate the growth of cancer cells (Rajivgandhi et al., 2022). The chemical compounds Hibisceusones A–C derived from *Hibiscus tiliaceus* effectively inhibited the activity of the phosphoinositide 3‐kinase‐α (PI3Kα) pathway, a critical signaling pathway involved in cellular growth (Chen et al., 2022). Several experiments revealed that the extract and separated compounds, Everninic acid and Roccellic acid, derived from *Phoenix paludosa* and *Roccella montagnei*, effectively inhibited cell growth (Mishra et al., 2017; Samarakoon et al., 2016). The molecule 3‐*O*‐methylthespesilactam, obtained from *Thespesia populnea*, has shown the ability to inhibit the phosphorylation of JAK1, JAK2, TYK2, and STAT3 signaling pathways. Additionally, it mediates cell cycle arrest, specifically during the S phase. In addition to inhibiting PARP, the compound also leads to the suppression of cancer cell growth and proliferation via the modulation of Mcl‐1 protein expression (Li et al., 2013). Cancer cell growth has been seen to be downregulated by the induction of cell death by the silver nanoparticles derived from *Caulerpa scalpelliformis* (Manikandan et al., 2019).

#### 3.3.7 Antimetastatic effect

Metastasis of cancer signifies a progressed phase of malignancy and is the primary contributor to mortality associated with cancer. Metastasis encompasses a series of sequential events involving the migration and invasion of cancer cells, which are recognized as fundamental characteristics of malignancy (Fares et al., 2020). The migration and invasion of cancer cells are closely linked to the generation of ROS, the emergence of chemotherapy‐resistant cancer stem cells, the occurrence of mutations in genes involved in DNA damage repair, and the involvement of microRNAs (miRNAs) (Bhuia et al., 2023a; Tahtamouni et al., 2019). Matrix Metalloproteinases‐2 and ‐9 (MMP2 and MMP9) are enzymes that facilitate the degradation of various components within the extracellular matrix. Consequently, these enzymes assume a critical function in cell migration in normal and pathological contexts (Cabral‐Pacheco et al., 2020). Likewise, vimentin facilitates cellular motility and initiates early cancer invasion through its transcriptional control of many genes and activation of numerous signaling pathways, including the Ras‐related protein Rab‐25 (RAB25)‐mediated protein kinase B (AKT)/glycogen synthase kinase‐3β (GSK‐3β)/Snail‐signaling pathway (Usman et al., 2021). The Snail family of zinc‐finger transcription factors includes Snail, the gene product of SNAI1. Snail induces epithelial–mesenchymal transition (EMT) by suppressing the expression of E‐cadherin. This process eventually promotes cancer invasion in several malignancies, including breast, hepatocellular, ovarian, cutaneous, and head and neck carcinomas (Cano et al., 2000; Yang et al., 2017). Antimetastatic medications exert their inhibitory effects on the migration and invasion of cancer cells by modulating gene expression, a process initiated by signal transduction pathways (Gandalovičová et al., 2017; Liu et al., 2020).

**FIGURE 5:**
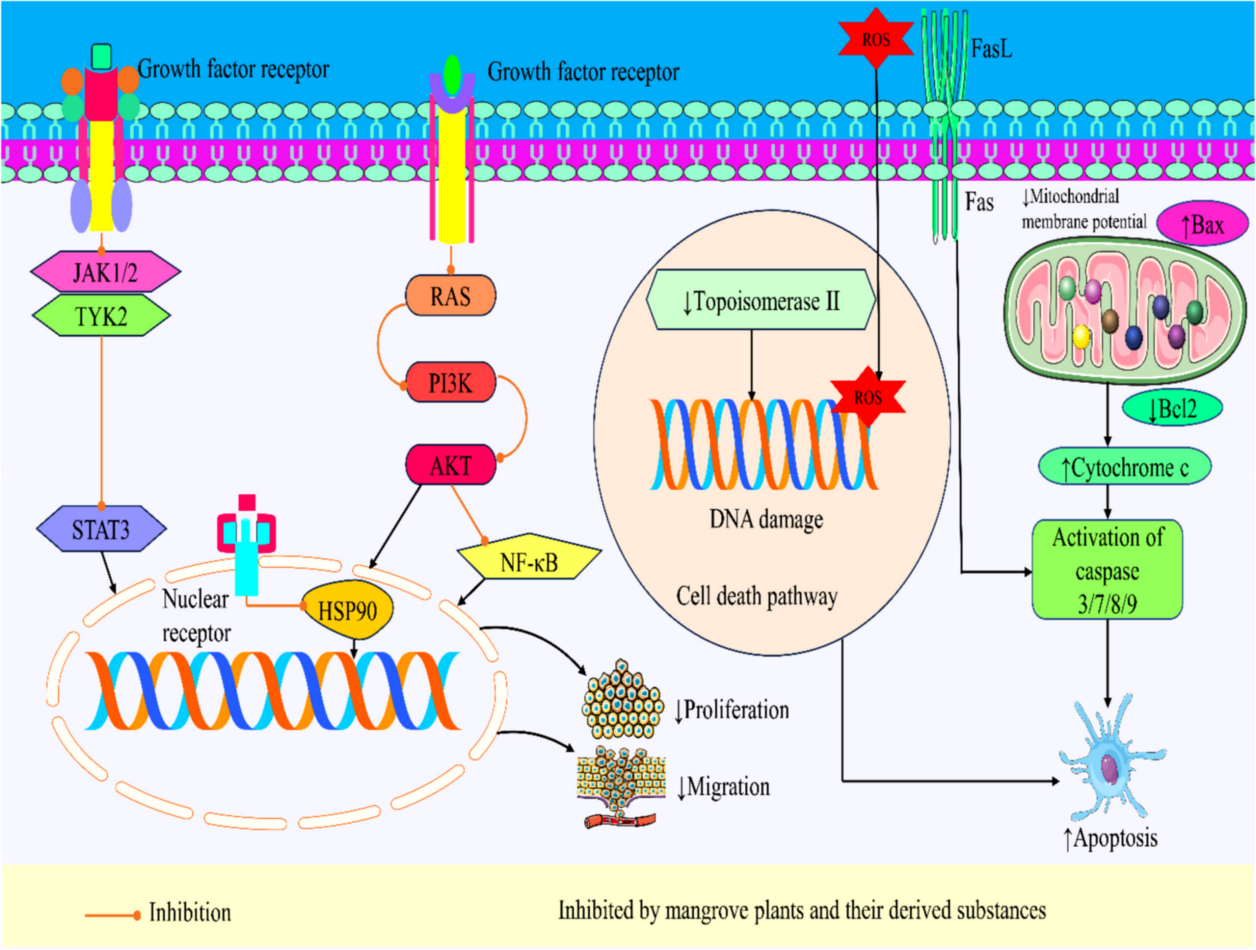
Possible oxidative bursts, antiproliferative, antimetastatic, and apoptotic mechanisms of mangrove plants and their derived compounds (AKT, protein kinase B; Bax, Bcl‐2‐associated X protein; Bcl‐2, B‐cell lymphoma 2; HSP90, heat shock protein 90; JAK1/2, Janus kinase 1/2; NF‐κB, nuclear factor kappa‐light‐chain‐enhancer of activated B cells; PI3K, phosphoinositide 3‐kinase; RAS, rat sarcoma (a family of small GTPases); ROS, reactive oxygen species; STAT3, signal transducer and activator of transcription 3; TYK2, tyrosine kinase 2).

Studies demonstrated that mangrove plants and their bioactive substances had antimetastatic capabilities, making them a promising candidate for investigating possible anticancer benefits. The leaf extract derived from the *Avicennia marina* mangrove plant blocks the migration of breast cancer cells by reducing the production or activity of MMP2, ‐9, PARP, vimentin, and snail proteins (Huang et al., 2016). Sahai et al. (2020) showed that Gedunin, derived from *Xylocarpus granatum*, could decrease the activity of PARP protein, resulting in elevated DNA damage and inhibition of the migratory process in ovarian cancer cells (Sahai et al., 2020). Additionally, the findings of other studies indicated that the extract of *Rhizophora apiculata* had inhibitory effects on the metastatic process in an in vivo test system (Prabhu & Guruvayoorappan, 2012, 2013). Figure 5 illustrates the possible oxidative bursts, antiproliferative, antimetastatic, and apoptotic mechanisms of mangrove plants and their derived compounds.

#### 3.3.8 Autophagy

Autophagy is a natural cellular process for breaking down and eliminating misfolded proteins and injured organelles that adapt to famine, development, cell death, and tumor suppression (Yun & Lee, 2018). Stress, like hypoxia, nutrient deprivation, or contact with a cytotoxic substance, could cause autophagy to be triggered (Dong et al., 2019; Kroemer et al., 2010; Packer, 2022). Several proteins regulate the autophagic process. A substrate for autophagy called p62 (Sequestosome‐1) is employed as a reporter of autophagic activity. The study also demonstrated that p62 transports ubiquitinated proteins, such as tau, to the proteasome for destruction (Chen, Li, Li, et al., 2020a; Liu et al., 2016). Under stressful circumstances, a lack of ribosomal protein S6 (S6) kinase causes a buildup of autophagosomes and a decrease in autophagolysosomes, affecting the autophagy flux. Additionally, a deficiency of S6 kinases lowers cell survival (Hać et al., 2015; Ravikumar et al., 2010). Cell proliferation, stress, and cancer development are linked to mammalian target of rapamycin (mTOR) (El‐Tanani et al., 2023; Gibbons et al., 2009; Kim & Guan, 2015). AMP‐activated protein kinase (AMPK) controls mTOR; thus, increasing AMPK and decreasing mTOR promote autophagy (Din et al., 2012). The Unc‐51‐like autophagy‐activating kinase (ULK) complex is dephosphorylated and activated when mTOR is inhibited (Lin & Hurley, 2016; Park et al., 2016). Many proteins involved in the development and extension of the autophagosome are recruited by Beclin‐1 (Bcl‐2‐interacting protein) (Kang et al., 2011; Pattingre et al., 2008). Autophagy‐related genes (ATGs) exert regulatory control over the elongation phase of autophagosome formation. The recruitment of microtubule‐associated protein 1 light chain 3 (LC3) is facilitated by autophagy‐related 5–autophagy‐related 12/autophagy‐related 16 (ATG5–ATG12/ATG16) complexes, which are also related to the expansion of phagophores (Li & Zhang, 2019; Lippai & Lőw, 2014; Varga et al., 2022). Next, LC3 fuels phagophore elongation, and autophagy‐related 4 (ATG4) transforms pro‐LC3 into the cytosolic isoform of LC3 that is active (Sekar & Thirumurugan, 2022; Yun & Lee, 2018). Microtubule‐associated protein 1A/1B‐light chain 3‐I (LC3‐I) subsequently interacts with phosphatidylethanolamine (PE), autophagy‐related 3 (ATG3), and autophagy‐related 7 (ATG7), converting into microtubule‐associated protein 1A/1B‐light chain 3‐II (LC3‐II). The autophagosome possesses inner and outside membranes enriched in LC3‐II, facilitating its interaction with substrates that have undergone degradation (Kabeya et al., 2000; Raudenska et al., 2021). Autolysosomes are generated through the fusion of mature autophagosomes with lysosomes, resulting in the formation of autophagolysosomes. These cellular structures employ the process of autophagy to eliminate faulty proteins and damaged organelles (Uddin et al., 2019).

**FIGURE 6:**
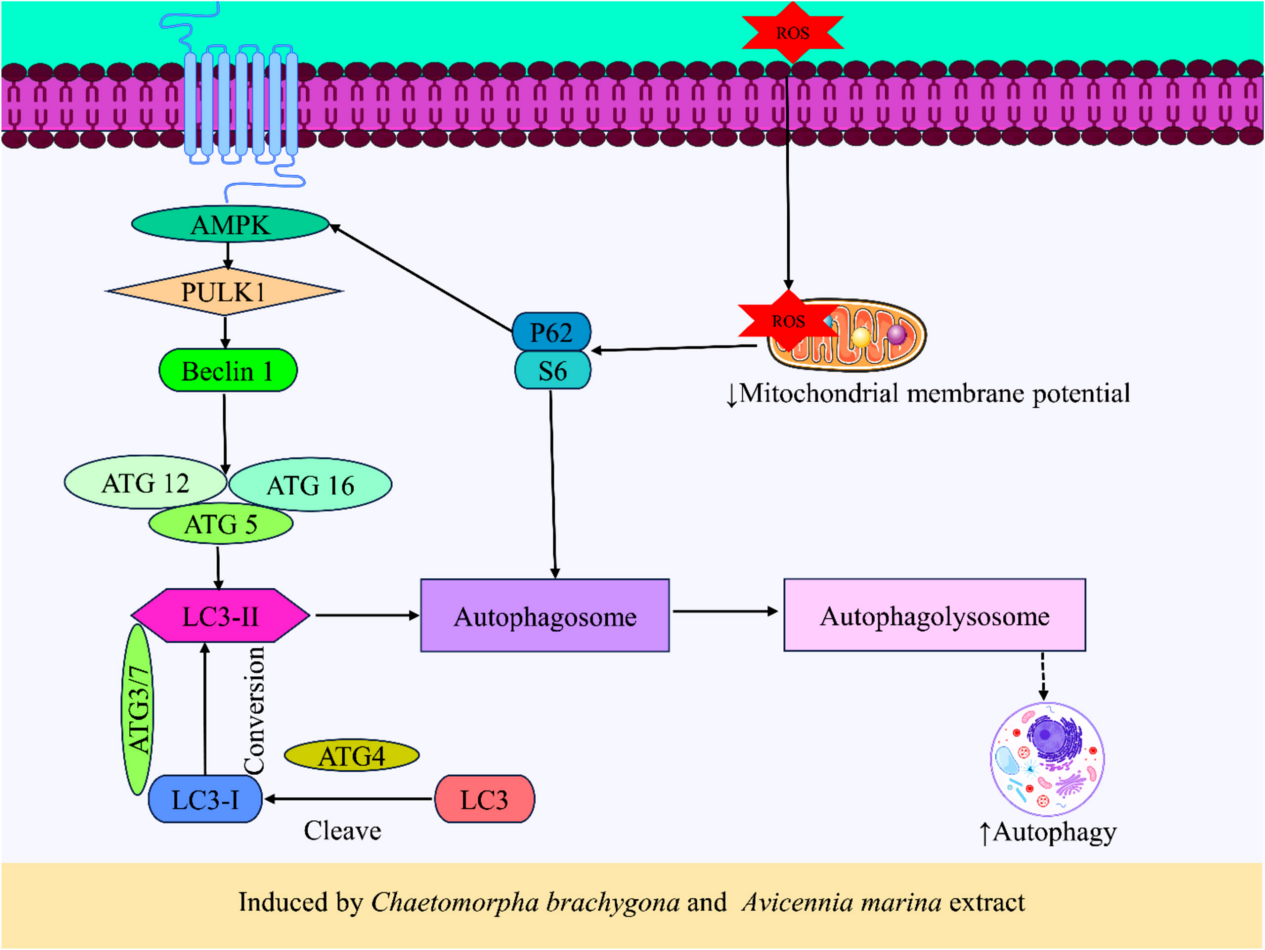
Possible autophagy effects of mangrove plants and their derived compounds (AMPK, AMP‐activated protein kinase; ATG12, autophagy‐related 12; ATG16, autophagy‐related 16; ATG3/7, autophagy‐related 3/7; ATG4, autophagy‐related 4; ATG5, autophagy‐related 5; Beclin 1, Bcl‐2‐interacting protein; LC3‐I, microtubule‐associated protein 1A/1B‐light chain 3‐I; LC3‐II, microtubule‐associated protein 1A/1B‐light chain 3‐II; P62, sequestosome‐1; ROS, reactive oxygen species; S6, ribosomal protein S6; ULK1, Unc‐51‐like autophagy activating kinase 1).

Several investigations have revealed that mangrove plants induce autophagy in cancer treatment. A mangrove plant extract of *Avicennia marina* promotes autophagy and controls cancer proliferation via induced ROS production (Esau et al., 2015). Another study demonstrated that the extract of *Chaetomorpha brachygona* has accelerated ROS production and increased the activation of AMPK, Beclin 1, S6, p62, and the Microtubule‐associated protein light chain 3 (LC3BII) autophagy regulator protein in cervical cancer cells. In the same study, the extract also decreased the mitochondrial membrane potential, promoting autophagy (Majumder et al., 2020). Figure 6 illustrates the autophagy mechanism of mangrove plants.

#### 3.3.9 Miscellaneous effects

Evidence suggests that lipid peroxidation is correlated with an increased susceptibility to cancer development (Gago‐Dominguez et al., 2007). Malondialdehyde (MDA) is a byproduct arising from polyunsaturated fatty acids' peroxidation in cellular environments. The overproduction of MDA results from an elevated presence of free radicals (Gaweł et al., 2004). The MDA level is well recognized as a biomarker for evaluating oxidative stress and antioxidant status in individuals with cancer (Didžiapetrienė et al., 2020). Glutathione (GSH) plays a remarkable role in the control of carcinogenic pathways, susceptibility to cytotoxic medicines, ionizing radiation, and some cytokines, as well as DNA synthesis, cell proliferation, and cell death in cancer cells (Ortega et al., 2011; Traverso et al., 2013). Gamma‐glutamyl transpeptidase (GGT) expression is often markedly elevated in several types of human malignancies (Bayrak et al., 2022). Nitric oxide (NO) and its associated reactive nitrogen species (RNS) have potential genotoxic and angiogenic characteristics. Elevated production of NO within a cellular environment can favor the survival and proliferation of mutant p53 cells, hence facilitating the process of tumor angiogenesis via the upregulation of vascular endothelial growth factor (Xu et al., 2002). Anticancer medications inhibit the biomarker antioxidant proteins, which prevent the proliferation of cancer cells (Greenwell & Rahman, 2015).

**FIGURE 7:**
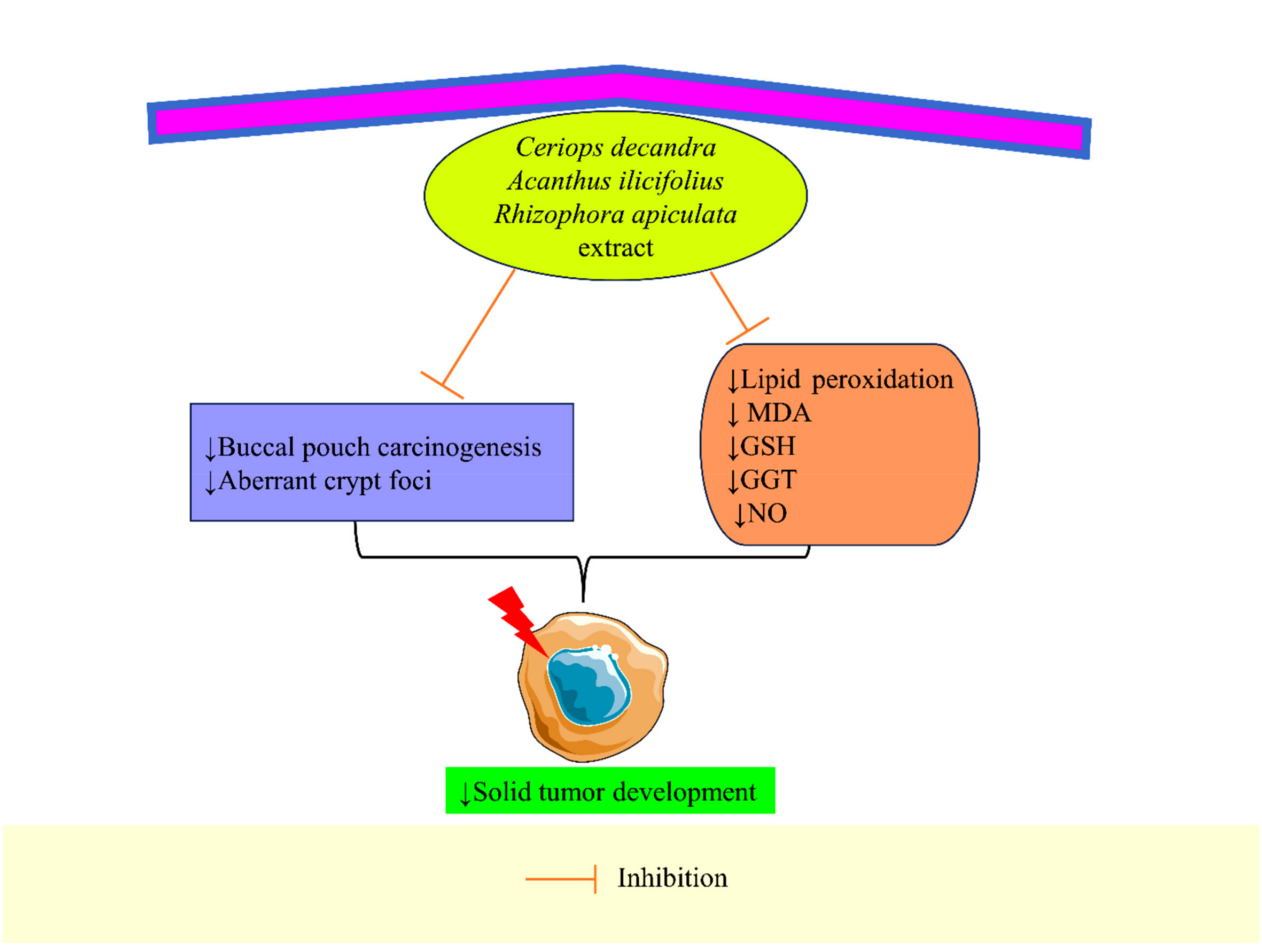
Miscellaneous anticancer mechanisms of mangrove plants and their derived compounds (GGT, gamma‐glutamyltransferase; GSH, glutathione; MDA, malondialdehyde; NO, nitric oxide).

The mangrove plants and their derived phytochemicals have anticancer properties. The extract made from the mangrove plant *Rhizophora apiculata* has shown a reduction in the formation of solid tumors by decreasing the levels of GSH, GGT, and NO molecules (Prabhu & Guruvayoorappan, 2012, 2013). The study of *Acanthus ilicifolius* extract shows that it effectively inhibits cell proliferation and aberrant crypt foci development, by attenuating lipid peroxidation and the activity or amount of MDA (Almagrami et al., 2014). Another study by (Boopathy et al., 2011) showed that the administration of *Ceriops decandra* extract reduced buccal pouch carcinogenesis (Boopathy et al., 2011). Miscellaneous anticancer mechanisms of mangrove plants and their derived compounds are illustrated in Figure 7.

### 3.4 Pharmacokinetics of mangrove plants' active phytochemicals

The pharmacological efficacy and potency of a medication within the body are intricately linked to its processes of absorption, distribution, metabolism, and excretion (Hurst et al., 2007). Consequently, pharmacokinetic properties play a crucial role in the development of drugs for specific diseases and conditions. Mangrove plants and their active compounds have demonstrated anticancer properties, underscoring the importance of in‐depth pharmacokinetic studies for the development of these phytochemicals as anticancer agents.

In female CD‐1 strain mice, the mangrove‐active phytochemical lupeol at 200 mg/kg showed rapid absorption and good oral bioavailability. Pharmacokinetic parameters in plasma were calculated using the mono‐compartmental model. The half‐life (*t*_1/2_), area under curve (AUC(0–*t*)), peak plasma time (*T*_max_), peak plasma concentration (*C*_max_), volume of distribution (*V*_d_), and clearance (CL) were 13.564 ± 2.912 h, 132.530 ± 29.850 μg × h/mL, 6.444 ± 0.851 h, 8.071 ± 2.930 μg/mL, 595.902 ± 210.773 L, and 29.870 ± 4.596 L/h, respectively. The compound was primarily eliminated, with a maximal elimination time of 12 h and a value of 163.28 ± 9.83 ng/mg (Cháirez‐Ramírez et al., 2019). A similar pharmacokinetic study of lupeol after intravenous (1 mg/kg) and oral (30 mg/kg) administration in rats revealed that the bioavailability of the oral route was less than 1%. Therefore, different formulation approaches need to be evaluated in the future to enhance the solubility and oral bioavailability of lupeol (Khatal & More, 2019). Furthermore, the pharmacokinetic results indicated that lupeol‐loaded PEGylated liposomes increase AUC by 3.2 times compared to free lupeol after intravenous (i.v.) administration, with mean residence time (MRT) and *t*_1/2_ values showing 2.5 and 4.1 times improvements, respectively (Zhang et al., 2019). Additionally, bioavailability studies of *Ficus religiosa* L. extract in suspension and solid lipid nanoparticle (SLN) forms demonstrated that SLN increased lupeol's AUC by 9.2‐fold and *C*_max_ by 3.9‐fold compared to suspension, while prolonging *t*_1/2_ from 7.3 ± 1.0 to 15.3 ± 1.3 h. These findings suggest that SLN enhances AUC and *C*_max_ while prolonging *t*_1/2_, potentially enabling dose reduction, prolonged action, and enhanced therapeutic efficacy (Priyanka et al., 2017). A flavonoid mangrove compound, luteolin, exhibited poor oral bioavailability, primarily appearing in plasma as glucuronides and sulfate conjugates after metabolism while also displaying potent inhibition of OATP1B1 (organic anion transporting polypeptide 1B1) and OATP2B1 (organic anion transporting polypeptide 2B1). This characteristic may result in pharmacokinetic interactions with other bioactive substances (Shi et al., 2024). Likewise, the pharmacokinetic analysis of luteolin revealed a *t*_1/2_ (8.94 h) for free and a *t*_1/2_ (4.98 h) for conjugated luteolin after intravenous administration. Following oral administration, luteolin reached a *C*_max_ of 5.5 μg/mL at 5 min, declining below the limit of quantification after 1 h, with a low bioavailability of 4.10% attributed to a significant first‐pass effect (Sarawek et al., 2008). Another study revealed that oral administration of quercetin had low bioavailability and was limited in crossing the blood–brain barrier (BBB). However, when quercetin was combined with lipid nanoparticles, its ability to traverse the BBB and enter the brain was substantially enhanced (Chowdhury et al., 2023). A comprehensive study evaluating the pharmacokinetics and enterohepatic recirculation of quercetin in humans revealed that it had a high oral CL of 3.5 × 10^4^ L/h, with an average terminal *t*_1/2_ of 3.5 h. Plasma concentration versus time curves exhibited reentry peaks, which were best described by a one‐compartment model incorporating enterohepatic recirculation (Moon et al., 2008). Another mangrove compound, patriscabratine, exhibited metabolic stability in mouse, rat, and human liver microsomes, with t_1/2_ durations of 3.90, 3.93, and 5.48 min, respectively (Mak et al., 2022). Pharmacokinetic studies on active compounds from mangrove plants are generally not comprehensive. Most studies revealed the limited bioavailability of these compounds upon oral administration. To address this issue, formulation improvement strategies, such as nanosuspensions, solid lipid nanoparticles, polymeric nano‐ and microparticles, phytosomes, liposomes, niosomes, and microemulsions, are recommended.

### 3.5 Toxicological profile of mangrove plants and their active phytochemicals

Toxicity testing encompasses a methodical assessment of the harmful physiological and biochemical consequences that a substance has the potential to induce (Anderson & Henck, 1994). The principal aim of toxicology investigations within the drug development phase is to assess the safety profile of prospective drug candidates (Dorato & Buckley, 2007). The prevalence of drug‐induced toxicities in the brain, heart, liver, and kidney has become a significant factor contributing to over 70% of drug attrition and withdrawal (Fang & Eglen, 2017). Preclinical toxicity testing is conducted on several biological systems to ascertain the harmful effects of an experimental substance, which are particular to the species, organ, and dosage (Parasuraman, 2011). The assessment of drug toxicity can be conducted by several methodologies, including in silico analysis (Wu et al., 2020), in vivo experimentation on animal models (Ruggeri et al., 2014), and in vitro investigations utilizing organ‐on‐a‐chip technology (Cong et al., 2020).

Toxicity assessment of mangrove plants and their active phytochemicals is crucial for the development of drugs for the particular disease. Research findings indicated that mangrove plants and their active phytochemicals have undergone limited toxicological studies. According to the research findings, the extracts of *Aegiceras corniculatum*, *Ceriops decandra*, *Avicennia officinalis*, *Nypa fruticans*, *Heritiera fomes*, and *Phoenix paludosa* fruits exhibited cytotoxic effects in brine shrimp lethality tests (BSLT) with LC_50_ (lethal concentration 50) values of 524.00, 116.70, 497.00, 299.30, 74.10, and 331.40 μg/mL, respectively (Hosen et al., 2021). Another study showed that the administration of *Avicennia marina* leaf extract did not result in significant alterations in the body and liver weights, fecal, water intake and urine output, erythrocyte counts and leukocytes, hemoglobin levels, or hematocrit levels. A notable reduction in platelet counts and a corresponding elevation in neutrophil counts were observed in rats (Ali & Bashir, 1998). In the study conducted by Das et al. (2019), the ethanolic bark extract of *Xylocarpus granatum* (1000 mg/kg) was administered orally to healthy BALB/c mice and demonstrated no indications of toxicity or mortality with a duration of up to 4 days (Das et al., 2019). Furthermore, the ethyl acetate extract of *Xylocarpus granatum* leaves showed no significant toxicity to the BSLT with an LC_50_ value above 1500 ppm (parts per million) (Darmadi et al., 2021). Additionally, the ethanolic extracts of the aerial roots of *Ceriops decandra* and *Ceriops tagal* (250–1000 mg/kg) showed no acute toxicity, mortality, or behavioral alterations, as well as a slight increase in the mice's weight after 14 days of treatment (Biswas et al., 2021). The aqueous extract of *Ceriops tagal*, at concentrations ranging from 5 to 50 mg/mL, demonstrated no toxicity in experimental animals. However, over a study period of 7 days, when exposed to a concentration of 60 mg/mL of *Ceriops tagal*, only 77.7% of the animals were found to have survived (Sudheer et al., 2012). Furthermore, the bark extract of *Rhizophora mucronata* at different concentrations of 800–3200 mg/kg showed no mortality or behavior change in healthy Swiss Albino mice during 14 days (Chitra et al., 2020). A different study revealed that, during the 14‐day period, administration of the methanolic extract of *Rhizophora mucronata* at a dosage of 2000 mg/kg did not result in any observable deleterious effects on the general appearance or mortality of mice. There was no statistically significant alteration in the increase in body weight reported among the groups treated with the extract (Suganthy et al., 2016). The methanolic extract of *Aegiceras corniculatum* (2000 mg/kg) showed no indication of toxicity. While at concentrations of 200 mg/kg, the extract elevated some parameters, such as aspartate transaminase (AST), alanine transaminase (ALT), alkaline phosphatase (ALP), acid phosphatase (ACP), triglyercide (TGL), high‐density lipoprotein (HDL), low‐density lipoprotein (LDL), and very‐low‐density lipoprotein (VLDL) in subacute toxicity tests in rats (Ravikumar et al., 2011). The proportion of ethyl acetate in latex derived from *Excoecaria agallocha* was acquired, demonstrating minimal irritating activity. Nevertheless, the active phytochemicals demonstrated negligible irritating effects on the mouse ear (Karalai et al., 1994). Further study revealed that the methanolic extract of *Acanthus ilicifolius* showed cytotoxicity against brine shrimp nauplii with an LC_50_ value of 22 μg/mL (Firdaus et al., 2013). The toxicity of *Cerbera odollam* has similarities to the acute poisoning caused by digoxin. The consumption of the kernel induces symptoms, such as nausea, vomiting, thrombocytopenia, hyperkalemia, and electrocardiogram (ECG) abnormalities. The greatest danger of mortality is associated with exposure to high doses (1.8–3.8 mg/kg) of *Cerbera odollam* (Menezes et al., 2018). Several studies demonstrated that the active phytochemicals (Cerberin and Neriifolin) of *Cerbera manghas* and *Cerbera odollam* induced heart failure by inhibiting the Na^+^/K^+^ATPase pump (Hossan et al., 2019; Tsai et al., 2008; Wermuth et al., 2018). The in vitro cytotoxicity of N‐*trans*‐feruloyltyramine, N‐*cis*‐feruloyltyramine, and Hibiscusamide bioactive compounds derived from *Hibiscus tiliaceus*, was observed against HT‐29 and P‐388 cell lines. The IC_50_ values for these substances were found to be less than 4 μg/mL (Chen et al., 2006). The results of acute toxicity studies revealed that oral treatment of *Thespesia populnea* extracts did not elicit any hazardous manifestations in mice. The LD_50_ value of the extract was found to be 2000 mg/kg of body weight. By convention, the ED_50_ (median effective dose) was typically one‐tenth of the LD_50_ value, thus indicating an ED_50_ of 200 mg/kg (Belhekar et al., 2013). In summary, a number of studies suggest mangrove plants have no toxicity, whereas some plants and their active phytochemicals show severe toxicity to organs, so it is imperative to conduct comprehensive trials in order to definitively ascertain the toxicity levels or upper tolerated limits.

### 3.6 Clinical evidence

Clinical trials are research studies conducted on human subjects to investigate biological or behavioral interventions, such as novel therapies or existing interventions that need more examination and comparison (Kruizinga et al., 2019). Clinical trials play a crucial role in the exploration of novel therapies for illnesses (Akhondzadeh, 2016). Drug and medical device clinical studies go through many stages to assess safety, establish efficacy, and detect any adverse effects (Friedman et al., 2015; Latha et al., 2020).

There is no clinical evidence of mangrove plants as anticancer agents. Mangrove plants have several classes of phytochemicals. Those classes of phytochemicals have shown an anticancer effect in different clinical studies. The clinical study of flavonoids (quercetin and luteolin) reduced ovarian cancer risk in women (Gates et al., 2007). Similarly, alkaloids, saponins, glycosides, terpenes, and polyphenols have shown anticancer effects in different types of cancer, including breast cancer, lung cancer, liver cancer, colon cancer, prostate cancer, and ovarian cancer (Chadid et al., 2022; Chen, Li, Yang, et al., 2020b; Douillard et al., 2006; Nuñez‐Sánchez et al., 2014; O'Shaughnessy et al., 2022; Winterhoff et al., 2015). In summary, a number of preclinical studies, both in vitro and in vivo, on mangrove plants have revealed positive outcomes in cancer treatment. Therefore, the clinical study of mangrove plants and their active phytochemicals is required.

Translational research, also known as translational medicine or translational science, is the process of applying knowledge gained from basic scientific research to clinical research. It aims to develop new treatments, medical devices, procedures, preventions, and diagnostics by bridging the gap between basic research and clinical applications (Cohrs et al., 2015). The translation of preclinical discoveries into clinical applications in the field of oncology can encounter numerous restrictions and challenges: (1) Cancer heterogenicity: Many drugs showed effectiveness in preclinical studies but failed in clinical studies, due to cancer heterogeneity. Cancer heterogeneity refers to the variations seen among tumors of the same kind in different people, among cancer cells within a single tumor, or between a primary tumor and a secondary tumor. These disparities may include the genetic and protein composition of the tumor (Denison & Bae, 2012). (2) Inadequately validated targets: The conventional approach of discovering genes in a laboratory setting and then creating animal models to study human diseases has proven to be difficult due to the frequent failure of animal‐based targets and therapies in human trials. As to the National Institutes of Health (NIH), around 80%–90% of research studies do not go to human testing and fail at earlier stages (Seyhan, 2019). (3) Efficacy and toxicity: The major cause of preclinical drug failure in clinical trials continues to be the inability to show effectiveness (Fogel, 2018). Simultaneously, animal toxicity testing is ineffective in predicting the toxicity of over 50% of medicines in the pipeline during Phase I studies. The translation of novel drug candidates from preclinical research to human studies and the subsequent approval rate is only roughly 0.1%. Most initiatives fail owing to issues that are not connected to a therapeutic premise, such as unforeseen side effects and tolerance (DiMasi et al., 2010; Van Norman, 2020). (4) Pharmacokinetics: Preclinical studies demonstrate the effectiveness of targeting tumors in animal models; however, the performance of the medicine in people may be considerably affected by pharmacokinetic difficulties (McGonigle & Ruggeri, 2014). Furthermore, financial constraints, patient recruitment and retention, and appropriate trial design are the primary obstacles to translating preclinical studies into clinical studies (Fogel, 2018; Hu, 2024).

## 4 CONCLUSION

To date, many studies have demonstrated the anticancer or antitumor effects of mangrove plants and their derived chemicals via modulation of various anticancer mechanisms, including oxidative stress and mitochondrial dysfunction, cytotoxicity, genotoxicity, cell cycle arrest, apoptosis, autophagy, antiproliferative, antimetastatic, and other miscellaneous actions. These suggest their potential as prospective candidates for the application of anticancer treatment for various cancers, such as breast, lung, liver, bone, colon, and cervical cancers, as well as solid tumors. The findings also demonstrated that mangrove plants and their derived compounds exerted anticancer potential by regulating various cellular pathways, such as JAK2/STAT3, NF‐κB, PI3K/AKT, and ERK1/2 (extracellular signal‐regulated kinase 1/2) signaling pathways. However, it is necessary to conduct more experiments that include a comprehensive analysis of various pharmacokinetic and toxicity characteristics to establish the viability of mangrove plants and their derived compounds as authorized therapeutic agents for cancer therapy. Hence, more investigations are required on the toxicity assessments of mangrove plants and the compounds generated from them prior to embarking on comprehensive clinical trials. In the foreseeable future, the potential of mangrove plants and their derived compounds as very effective chemotherapeutic medicines is expected to be significantly enhanced after successful validation in human clinical studies. In conclusion, this research elucidates the potential of mangrove plants and their derived compounds as valuable adjunctive therapies for the prevention and treatment of many types of cancer. Additionally, it highlights their potential as a promising framework for advancing novel anticancer agents in the foreseeable future.

## AUTHOR CONTRIBUTIONS

**Raihan Chowdhury:** Data curation (equal); investigation (equal); methodology (equal); visualization (equal); writing – original draft (equal); writing – review and editing (equal). **Md. Shimul Bhuia:** Data curation (equal); investigation (equal); methodology (equal); validation (equal); writing – original draft (equal); writing – review and editing (equal). **Md. Sakib Al Hasan:** Data curation (equal); investigation (equal); methodology (equal); validation (equal); visualization (equal); writing – original draft (equal); writing – review and editing (equal). **Shadid Hossain Snigdha:** Data curation (equal); investigation (equal); methodology (equal); visualization (equal); writing – original draft (equal); writing – review and editing (equal). **Sadia Afrin:** Data curation (equal); investigation (equal); methodology (equal); visualization (equal); writing – original draft (equal); writing – review and editing (equal). **Dietrich Büsselberg:** Data curation (equal); investigation (equal); methodology (equal); supervision (equal); validation (equal); visualization (equal); writing – original draft (equal); writing – review and editing (equal). **Solomon Habtemariam:** Data curation (equal); investigation (equal); supervision (equal); validation (equal); visualization (equal); writing – original draft (equal); writing – review and editing (equal). **Eda Sönmez Gürer:** Data curation (equal); investigation (equal); methodology (equal); writing – original draft (equal); writing – review and editing (equal). **Afaf Ahmed Aldahish:** Data curation (equal); investigation (equal); methodology (equal); writing – original draft (equal); writing – review and editing (equal). **Javad Sharifi‐Rad:** Data curation (equal); investigation (equal); methodology (equal); project administration (equal); supervision (equal); validation (equal); visualization (equal); writing – original draft (equal); writing – review and editing (equal). **Nursulu Аkhtayeva:** Data curation (equal); investigation (equal); methodology (equal); writing – original draft (equal); writing – review and editing (equal). **Muhammad Torequl Islam:** Conceptualization (equal); data curation (equal); investigation (equal); methodology (equal); project administration (equal); resources (equal); supervision (equal); validation (equal); visualization (equal); writing – original draft (equal); writing – review and editing (equal).

## CONFLICT OF INTEREST STATEMENT

The authors wish to confirm that there are no known conflicts of interest associated with this publication, and there has been no significant financial support for this work that could have influenced its outcome.

## Data Availability

The authors have nothing to report.
